# Pore graded borosilicate bioactive glass scaffolds: in vitro dissolution and cytocompatibility

**DOI:** 10.1007/s10856-024-06791-1

**Published:** 2024-03-20

**Authors:** Agata Szczodra, Amel Houaoui, Turkka Salminen, Markus Hannula, Virginia Alessandra Gobbo, Sonya Ghanavati, Susanna Miettinen, Jonathan Massera

**Affiliations:** 1 Tampere University, Faculty of Medicine and Health Technology, Tampere, Finland; 2 Tampere University, Faculty of Engineering and Natural Sciences, Tampere, Finland; 3 Research Services, Wellbeing Services County of Pirkanmaa, Tampere University Hospital, Tampere, Finland

## Abstract

3D borosilicate bioactive glass (1393B20 and B12.5MgSr) scaffolds were prepared by robocasting, with and without a dense layer at the top. Pore graded scaffolds are promising as they allow for membrane deposition and could limit the risk of soft tissue infiltration. In vitro dissolution was studied in tris(hydroxymethyl)aminomethane (TRIS) and Simulated Body Fluid (SBF). 1393B20 scaffolds dissolved faster than B12.5MgSr in TRIS whereas they dissolved slower in SBF. The difference in dissolution profiles, as a function of the medium used, is assigned to the different rates of precipitation of hydroxyapatite (HA). While the precipitation of calcium phosphate (CaP) in the form of HA, first sign of bioactivity, was confirmed by ICP, FTIR-ATR and SEM-EDX analysis for both compositions, 1393B20 was found to precipitate HA at a faster rate. The presence of a dense top layer did not significantly impact the dissolution rate and CaP precipitation. In vitro cell culture was performed using human adipose-derived stem cells (hADSCs). Prior to cell plating, a preincubation of 3 days was found optimum to prevent burst ion release. In direct contact, cells proliferate and spread on the scaffolds while maintaining characteristic spindle morphology. Cell plated on 1393B20 scaffolds showed increased viability when compared to cell plated on B12.5MgSr. The lower cell viability, when testing B12.5MgSr, was assigned to the depletion of Ca^2+^ ions from culture medium and higher pH. Static cell culture leads to believe that the scaffold produced from the 1393B20 glass composition are promising in bone regeneration applications.

**Graphical Abstract:**

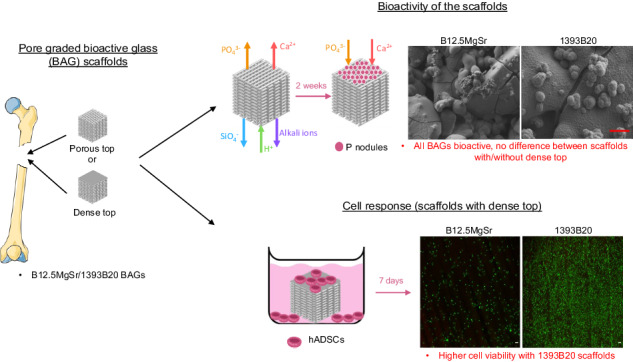

## Introduction

Due to high crystallization tendency during sintering process, there is no commercially available 3D porous bone graft made from silicate bioactive glass (BAG) [1–4]. Borosilicate BAGs demonstrate suppressed crystallization tendency during sintering and convert into HA faster and more completely compared to silicate BAGs [4–7]. Thus, researchers show continuous effort to develop the first commercial borosilicate 3D porous scaffolds. Moreover, presence of boron in glass composition can have a beneficial impact on cells differentiation and vascularization. Boron content between 1 and 100 ng/mL can stimulate osteogenic differentiation of human bone marrow-derived mesenchymal stem cells (hBMSCs) [8]. Similar observations were made with murine calvarial pre-osteoblastic cells (MC3T3-E1) and myoblastic cells (C2C12) [9, 10]. B_2_O_3_ substitution for SiO_2_ in S53P4 showed strong ability to stimulate osteogenic commitment and upregulate endothelial markers in human adipose-derived stem cells (hADSCs) [11]. Furthermore, boron released from the borosilicate BAGs stimulate angiogenesis of the embryonic quail chorioallantoic membrane in vivo [12]. The use of borosilicate glass would allow to obtain porous 3D scaffolds while improving the bioactivity. The requirements for 3D scaffolds are large pores (50–500 µm), highly interconnected porosity (> 50 µm) and overall porosity over 50% in order to provide proper osteoconduction, bone repair and vascularization [13, 14].

Here, two borosilicate glasses based on commercial bioactive glasses S53P4 (BonAlive) and 1393 are studied. By introducing boron, magnesium, and strontium into S53P4, the new B12.5MgSr composition was produced. Secondly, by introducing boron into 1393, new 1393B20 composition was produced.

Generally, the replacement of CaO with SrO and/or MgO, 1) helps to control the dissolution rate due to its stabilizing effect on borate network [15], 2) suppresses the crystallization tendencies during sintering by increasing the hot forming domain [16, 17], 3) promotes bone repair and remodelling [18–20]. Moreover, Mg is essential for bone development and homeostasis, and it has been shown to stimulate osteogenesis in human osteoblasts [21, 22]. Addition of Sr has been shown to promote the proliferation and differentiation of osteoblasts [23–25] and to stimulate an osteogenic response in hBMSCs [26, 27].

The thermal and in vitro dissolution properties of the considered BAGs were investigated in the past [15]. Crystallization mechanism and sintering ability of B12.5MgSr glass were investigated. It has been shown that sintering this glass at 550 ^°^C does not cause crystallization [28]. 1393B20 BAGs are based on 1393 BAGs which crystallization mechanics have been also investigated in the past [29, 30]. 1393 scaffolds were shown to be sintered without crystallization at 700–720 °C [31–34]. 1393B1 and 1393B3 scaffolds were sintered at 630 °C and 570 °C, respectively, and shown to be non-crystalline [34].

Based on these results, in a previous study of the authors, B12.5MgSr glasses were sintered into traditional glass scaffolds with a net-like structure [35]. Then, the process was also optimized for 1393B20 bioactive glass composition. However, to optimize the bone reconstruction, the scaffold should mimic more closely the bone structure. For instance, while the centre of the scaffold should be highly porous, the top surface of the scaffold would benefit of being dense, as seen for the cortical bone. Therefore, pore graded scaffolds were produced. The graded porosity, with dense layer at the top of the porous structure, would also allow for the deposition of a membrane to minimize soft tissue infiltration [36]. 3D printing technique allows a precise control over the object structures, such as interconnectivity, shape, orientation, and pore size which can be customized through ‘layer-by-layer’ manufacturing [37–39].

After 3D printing, porous scaffold’s microstructure and mechanical properties were characterized. Then, the dissolution of glasses was investigated in static conditions in TRIS. Moreover, scaffolds bioactivity was investigated in static in vitro dissolution in SBF using ICP, SEM-EDX and FTIR-ATR. The impact of preincubation on ions release profiles was also studied. The aim was to assess the preincubation time to prevent the burst release of ions that could lead to cell death. Then, the ability of different scaffold types and compositions to support cell attachment and proliferation was assessed.

The aim of the present study was to develop a borosilicate BAG scaffolds, with optimized structure, for bone tissue engineering.

## Materials and methods

### Preparation of BAG powders

1393B20 and B12.5MgSr were prepared from analytical grade Na_2_CO_3_, NH_4_H_2_PO_4_, CaCO_3_, MgO, SrCO_3_, H_3_BO_3_ (Sigma Aldrich, St Louis, MO, USA), K_2_CO_3_ (Alfa Aesar, Haverhill, MA, USA) and Belgian quartz sand. 1393B20 and B12.5MgSr were melted in 60 g batches in a platinum crucible for 30 min at 1450 °C and 1300 °C, respectively. Melting was done in air atmosphere in LHT 02/17 LB Speed electric furnace (Nabertherm GmbH, Lilienthal, Germany). The molten glasses were casted and annealed in electric muffle furnace (Nabertherm L 3/12). The glasses were annealed for at least 6 h at 500 °C.

The casted glasses were first crushed and milled using a planetary ball mill (Fritsch GmbH, Idar-Oberstein, Germany). Then powders were sieved (Gilson Company, Inc., Ohio, USA) to particle size < 38 µm. The nominal oxide compositions of the glasses are given in Table 1.

**Table 1:** Nominal glass composition (mol %)

	SiO_2_	B_2_O_3_	P_2_O_5_	Na_2_O	K_2_O	CaO	MgO	SrO
**1393B20**	43.68	10.92	1.7	6	7.9	22.1	7.7	0
**B12.5MgSr**	47.12	6.73	1.72	22.66	0	6.77	5	10

### Scaffold manufacturing

Firstly, pluronic solution was prepared by mixing Pluronic 127 (Sigma-Aldrich, CAS No. 9003-11-6) with distilled water in an ice bath, stirring it for at least 6 h until the solution turned clear. Two concentrations of Pluronic 127 solutions were prepared, 25 and 30 wt%. During the optimization of 1393B20 ink, 30 wt% Pluronic 127 solution allowed better viscosity for 3D printing compared to 25 wt% Pluronic 127 solution when printing B12.5Mg-Sr. The solutions were then stored at 4 °C.

To make the ink, the glass powder was mixed with Pluronic 127 solution in the ratio of 30:70 wt% respectively, using Vibrofix VF1 electrical shaker (IKA®-Labortechnic, Staufen, Germany) at 2500 rpm. Each batch was vortexed with at least 5 mixing-cooling cycles (30 s mixing + 30 s cooling in the ice bath) until the ink was homogenous and did not show any visible bubble. Finally, the ink was loaded into an Optimum® 3cc printing cartridge (Nordson EFD, Bedfordshire, England) and left stabilizing for 20 min at room temperature.

**Fig. 1:**
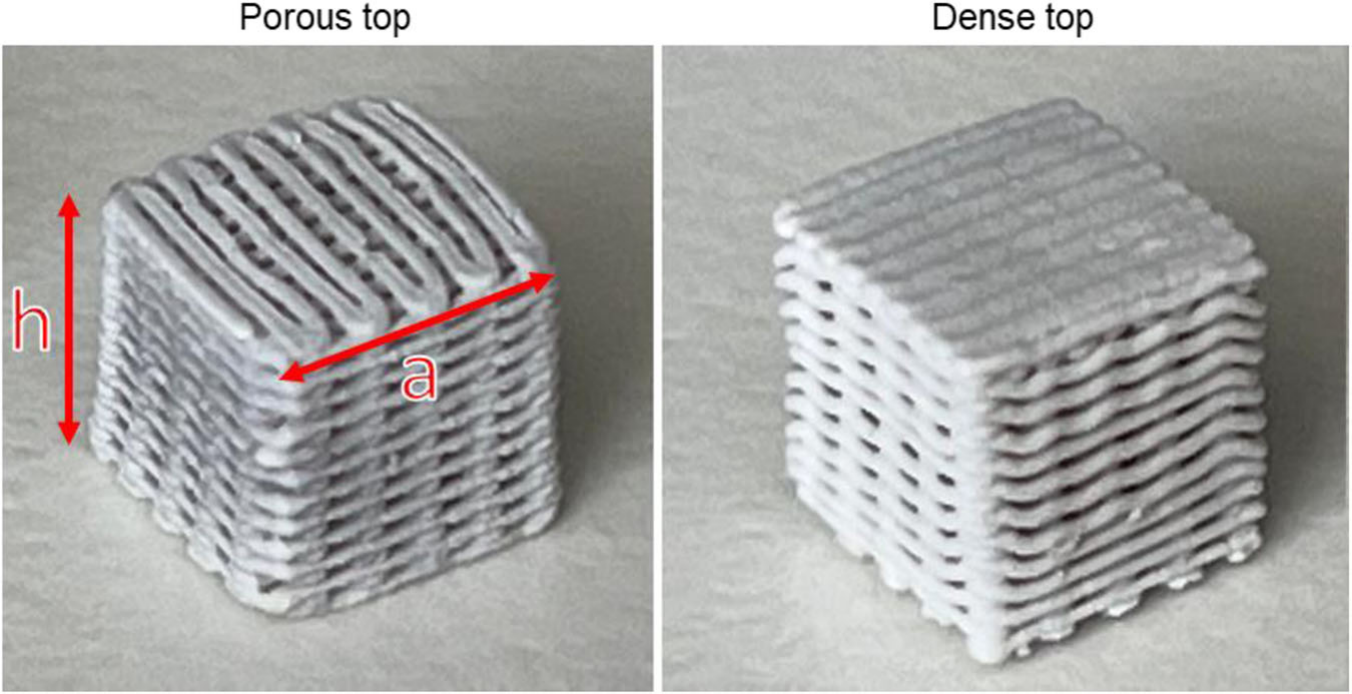
Image of the scaffolds with the porous top and dense top

3Dn-Tabletop printer (nScrypt Inc., Orlando, Florida, USA) controlled via the Machine Tool 3.0 system software was used for robocasting of 3D porous scaffolds. The cartridge was attached to the 3D printer and the ink extruded through the SmoothFlow Tapered Tips with a tip diameter of 0.41 mm (Nordson EFD Optimum® SmoothFlow™, Westlake, Ohio, USA). The ink was extruded onto an acrylic sheet (Folex AG, Seewen, Switzerland). The material feed was set to ~15.0–25.0 psi, to maintain a continuous flow during movement of the tip. The 3D printed scaffolds were made in cube shape with and without dense top. After drying at room temperature for at least 24 h to reduce the risk of collapse, scaffolds were sintered to allow fusing of glass particles and to remove the binder. B12.5MgSr and 1393B20 scaffolds were sintered for 1 h at 542 °C and 625 °C, respectively. Sintering was done in a furnace (Nabertherm LT 9/11/SKM electric muffle furnace) in an air atmosphere. Sintered scaffold dimensions were h ≈ 5.6 mm and a ≈ 6 mm in size (Fig. 1).

### Material characterization

#### X-ray powder diffraction (XRD)

XRD was used to evaluate if the 3D printed B12.5MgSr and 1393B20 scaffolds stayed amorphous after sintering. Scaffolds were ground to fine powder in a mortar. measurements were conducted in the 10–100° using 2θ diffraction angle range, using cobalt tube (K_α_ = 1.789 Å) with Empyrean (Malvern Panalytical, UK).

#### Micro-computed tomography (µCT)

Micro-computed tomography (µCT) was utilized to gain information about the scaffold 3D structures. Measurements were conducted with MicroXCT-400 (Carl Zeiss X-ray Microscopy, Inc., Pleasanton, California, USA) using 80 kV tube voltage and a 0.4x objective. The resulting pixel size was 24.2 µm. Porosity and pore size were calculated in ImageJ with BoneJ plugin. The visualizations were made in Avizo software (Thermo Fischer Scientific, Waltham, MA, USA).

#### Mechanical properties

Maximum compressive stress and Young’s modulus were tested using an Instron 4411 mechanical tester (Instron, Massachusetts, USA). The measurements were done until complete failure of the scaffold at a 0.5 mm/min deformation speed and using 2 kN load cell. To evaluate if the dense top layer has significant effect on mechanical properties, the measurements were done by applying force on 1) the top of the scaffold (perpendicular to the dense surface) and 2) on the side of the scaffold (parallel to the dense surface). The measurements were obtained from at least six parallel samples for each scaffold type and glass composition and expressed as mean ± standard deviation (SD).

### Physico-chemical characterization

#### Static in vitro dissolution in TRIS

3D printed scaffolds with dense and porous top made from 1393B20 and B12.5MgSr glass compositions were immersed in TRIS solution for up to 10 weeks in an incubator at 37 °C (Orbital incubator SI600, Stuart) with an orbital speed of 100 rpm. This was done to investigate the ion dissolution behavior in an environment where the ionic supersaturation is limited [40]. TRIS solution (50 mM) was prepared by mixing ultrapure TRIS (Sigma Aldrich, St Louis, MO, USA) and TRIS-HCl (Sigma Aldrich, St Louis, MO, USA) in deionized water. The pH of the solution was adjusted to 7.4 at 37 °C.

For the immersion of the samples, the volume of TRIS was adjusted to the mass of the scaffold to maintain a scaffold’s mass to volume ratio constant at 10 mg/ml. because it is the easiest, taking into consideration that glass with lower density has a higher surface ratio. Every week the immersion solution was refreshed to avoid oversaturation of the solution with the leached ions. At each timepoint (week 1, 2, 4, 6, 8, and 10), the pH of the immersion solution was measured at 37 °C using a S47-K SevenMultiTM pH-meter (Mettler-Toledo LLC, Ohio, USA). The ionic concentration was studied by Inductively Coupled Plasma – Optical Emission Spectroscopy (ICP-OES). After drying the samples for at least 24 h at 37 °C, the mass loss was calculated following the equation:

1 $${Mass}\,{loss}=\frac{{Wo}-Wt}{Wo}* 100$$

Where W_o_ is the original mass before immersion, and W_t_ is the dry mass post-immersion, at the immersion time t.

This study was conducted on three parallel samples and the results are presented as mean ± standard deviation (SD).

#### Bioactivity in SBF

Scaffolds in vitro bioactivity, which is described as the ability of the material to have dissolution by-products leading to the precipitation of a HA-like layer, was investigated in SBF. SBF, developed by Kokubo et al., was prepared following the methodology from the standard ISO/FDIS 23317 [41]. All scaffold types and compositions were immersed in SBF maintaining a mass/volume ratio constant at 10 mg/ml. Solution was not refreshed to allow oversaturation of ions and consequent precipitation of calcium phosphate. At each timepoint (1, 2, 3, 7 and 14 days), the pH of the solution was measured at 37 °C, the mass loss was calculated, and the ionic concentration was studied. This study was conducted on three parallel samples, and the results are presented as mean ± standard deviation (SD). A blank sample (SBF only) was also studied post-incubation to ensure the SBF stability over-time.

#### ICP analysis

The immersion solution collected from static in vitro dissolution in TRIS and SBF were diluted 10 times in 1 M high purity nitric acid for ion analysis. Inductively Coupled Plasma - Optical Emission Spectroscopy (ICP-OES) (Agilent technologies 5110, Santa Clara, CA, USA) was employed to quantify P (λ = 214.914 nm), Ca (λ = 422.673 nm), Mg (λ = 280.270 nm), Si (λ = 251.611 nm), B (λ = 249.772 nm), Sr (λ = 407.771 nm), K (λ = 766.491 nm) and Na (λ = 588.995 nm) concentrations in the immersion solutions. The analyses were conducted in triplicate and the results are presented as mean ± standard deviation (SD).

#### Structural properties by FTIR-ATR spectroscopy

To investigate the structural properties, B12.5MgSr and 1393B20 scaffolds (before and post-immersion in SBF) were first crushed into powder using a pestle and mortar. Next, Fourier transform infra-red (FTIR) spectroscopy was performed in Attenuated Total Reflectance (ATR) mode using a PerkinElmer Spectrum Two FTIR Spectrophotometer (PerkinElmer, Waltham, MA). The FTIR-ATR spectra were recorded in absorption modality once for each scaffold type within the range 450–4000 cm^−1^, background-corrected and normalized to the absorption band with the highest intensity at 910 cm^−1^.

#### Surface Analysis

To observe and analyse the composition of B12.5MgSr and 1393B20 scaffolds surface before and after incubation in SBF, Focused Ion Beam - Scanning Electron Microscopy (FIB/SEM) (Zeiss Crossbeam 540, Oberkochen, Germany) with energy-dispersive X-ray spectroscopy (EDX) was performed. Magnification of 2000× was used. The acceleration voltage used was 2.0 kV to limit the penetration depth and reduce (without suppressing completely) the signal from the unreacted glass under the reaction layer. For the EDX, the top of scaffolds was carbon coated.

### Cell analysis

#### hADSCs isolation and expansion

Human ADSCs were isolated as described previously [35, 42]. The isolated hADSCs were cultured in α-Minimum Essential Media (α-MEM) (Gibco, Life Technologies, Carlsbad, CA, USA) without nucleosides, supplemented with 5% human serum (Serana Europe, Germany GmbH) and 1% penicillin/streptomycin (Gibco, Life Technologies, Carlsbad, CA, USA). Cells were cultured at 37 °C in a humidified atmosphere of 5% CO_2_ balanced 95% air in incubator (Thermo Scientific forma steri-cycle i160 CO_2_) until they reached over 80% confluence.

#### Preincubation of scaffolds before cell test

All experiments with cells were done using the 3D printed scaffolds made out of B12.5MgSr and 1393B20 BAGs. Scaffolds with and without dense top does not show significant differences in bioactivity and mechanical properties (as described in 3.2 and 3.3). Consequently, only dense top scaffolds were used due to their improved applicability. These scaffolds were heat-sterilized for 3 h at 200 °C before preincubation. Then, scaffolds were preincubated for 48 h in TRIS and additional 24 h in αMEM (1% P/S) in incubator at 37 °C to decrease the initial burst release of ions from scaffolds that can potentially lead to cell death. The volume of TRIS and α-MEM used for preincubation was calculated to maintain a mass/volume ratio constant at 10 mg/ml. Preincubation time and solutions was optimized in our previous study [35] based on smaller B12.5MgSr scaffolds. Here, the test was repeated to confirm that 3 days of total preincubation time is also sufficient for bigger cubical scaffolds.

#### Live/dead assay

Live/Dead assay was used to investigate scaffolds cytotoxicity in direct contact with cells. Firstly, preincubated scaffolds were placed into 48-well plates (ThermoFisher Scientific, Waltham, MA, USA). Next, 25.000 hADSCs 1 ml of α-MEM culture medium (no glutamine, 5% human serum, 1% P/S) were cultured in direct contact with scaffolds for 1, 3 and 7 days. Culture medium was refreshed at day 2 and 4. The positive control used was the Tissue Culture Polystyrene (TCPS) 48-well plate.

At each timepoint, the cell culture medium was collected and diluted 10 times in ultrapure water for ICP-OES analysis. Next, the samples were rinsed using Dulbecco′s Phosphate Buffered Saline, DPBS (Gibco, Life Technologies, Carlsbad, CA, USA). Staining solution was prepared according to the Live & Dead Kit (Invitrogen, Life Technologies, Carlsbad, CA, USA), added to the wells and incubated for 30 min at room temperature. Viable and necrotic hADSCs cells were stained with 1% (v/v) of Calcein AM and 0.5% (v/v) Ethidium homodimer-1 solution. Finally, the samples were again rinsed with DPBS and cells were observed under the fluorescence microscope (Olympus IX51).

#### Cell proliferation

To quantitatively compare the viability of hADSCs cells on the different types of scaffolds, cell proliferation was studied using a CyQUANT Cell Proliferation Assay kit (Invitrogen, Life Technologies, Carlsbad, CA, USA). The human ADSCs cell seeding was done as described in section 2.5.3. Culture medium was refreshed at day 2 and 4. At each timepoint the cells were lysed with 500 µL 0.1% Triton X-100 (Sigma Aldrich, St Louis, MO, USA) buffer and conserved at −80 °C. After one freeze–thaw cycle, three 20 µL aliquots of each lysate were pipetted to a black nontreated 96-well plate (Fisher Scientific, Hampton, NH, USA) and mixed with 180 µL working solution containing CyQUANT GR dye and cell lysis buffer. The fluorescence at 520 nm was measured with a Spectrofluorometer VICTOR Nivo Multimode Microplate Reader (Perkin Elmer, USA).

Finally, GraphPad Prism 8 Software was used for statistical analysis of results. Statistical significance between different scaffold types was assessed by one-way analysis of variance (ANOVA). Statistical significance is taken for values of *p* < 0.005. The experimental results are expressed as means ± standard deviation (SD).

#### Morphology

The morphology of the cells on top of the scaffolds was observed after 1, 3 and 7 days of culture. 16.000 hADSCs in 1 mL of α-MEM culture medium (no glutamine, 5% human serum, 1% P/S) were cultured in direct contact with scaffolds. Culture medium was refreshed at day 2, 4 and 6. The control was 10 mm diameter glass coverslips (Marienfeld, Lauda-Konigshofen, Germany) in a 48-well plate.

At each time point, the cells were fixed with 4% (w/v) para-formaldehyde solution in PBS (AlfaAesar, Haverhill, MA, USA) for 15 min. Next, cells were permeabilized with 0.1% (v/v) Triton X-100 (Sigma Aldrich, St Louis, MO, USA) for 10 min. The nonspecific binding sites were blocked by incubating the scaffolds in Phosphate Buffered Saline (PBS) (Medicago AB, Uppsala, Sweden) containing 3% (wt/v) Bovine Serum Albumin (BSA, Sigma Aldrich, St Louis, MO, USA) for 45 min. Subsequently, the samples were incubated for 45 min with stains diluted in PBS. Cytoskeleton and nucleus were stained using FITC-labelled phalloidin (1:500) (Sigma Aldrich, St Louis, MO, USA P1951) and 4’,6-Diamidino-2-phenylindole dihydrochloride (1:2000) (DAPI, Sigma Aldrich, St Louis, MO, USA D9542), respectively. Finally, the samples were rinsed with PBS–BSA 0.5% and pure water and observed using a LSM800 confocal microscope (Zeiss, Iena, Germany).

## Results and discussion

### Micro-computed tomography (µCT)

**Fig. 2:**
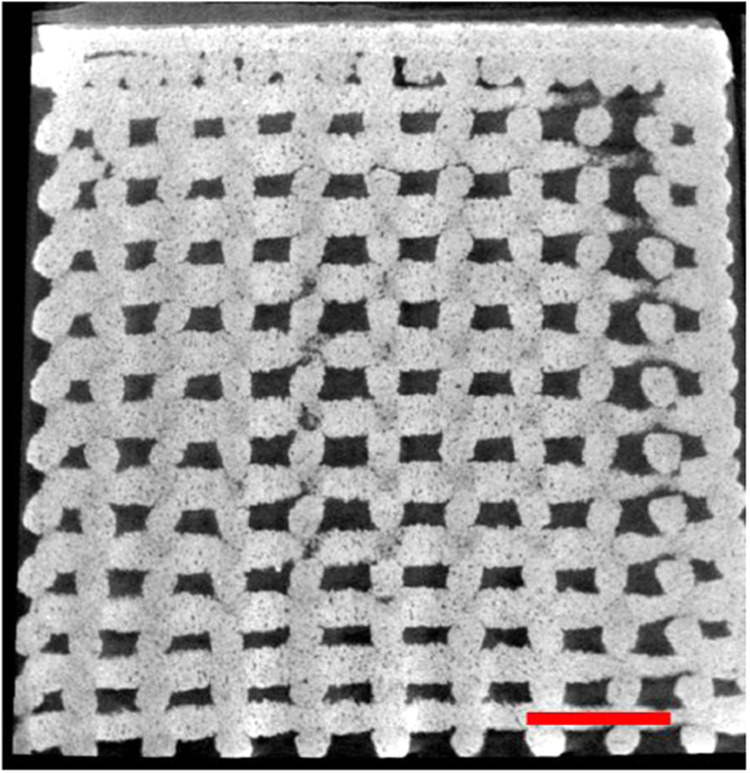
µCT image of the vertical cross-section of a scaffold with dense top, taken as an example. Scale bar 1 mm. The image is representative for both B12.5MgSr and 1393B20 glasses

Scaffolds microstructure have been investigated using µCT. As an example, an image of the vertical cross-section of a scaffold with a dense top is shown in the Fig. 2.

As visible in the image, the scaffold is built from parallel filaments with almost constant spacing that creates interconnected porosity. Due to shrinkage during sintering, the pores on the outside are smaller than in the core of the scaffold. The dense layer is comprised of the two top 3D printed layers and is relatively thin. Microstructure of all scaffolds were comparable and reproducible.

From the µCT images of the scaffold pre-immersion, the mean pore size and porosity were calculated and are presented in Table 2. The mean pore size and porosity is not significantly different between scaffold types and glass compositions. The mean pore sizes varied from ~277 to ~421 µm and are above the required size for the migration of cells as MC3T3-E1 cells or MSCs for example [43, 44]. The overall porosity for all scaffolds were between 43 and 46%.

**Table 2:** Mean pore size and porosity of B12.5MgSr and 1393B20 scaffolds with porous and dense top

		Mean pore size (µm)	Porosity (%)
B12.5MgSr	porous top	314 ± 140	46 ± 5
	dense top	321 ± 122	45 ± 5
1393B20	porous top	277 ± 103	45 ± 5
	dense top	282 ± 105	43 ± 5

Overall, the scaffolds were designed to meet the requirements for promoting cell migration. Scaffolds have large pores (50–500 µm), porosity close to 50% that is also highly interconnected to allow tissue infiltration and regeneration [13, 45]. The interconnected porosity also allows removal of waste and transport of nutrients and migration of cells inside the scaffolds [46]. Summarizing, robocasting has been shown to allow 3D printing of scaffolds with interconnected porosity made with both B12.5MgSr and 1393B20 BAG.

### Mechanical properties

**Fig. 3:**
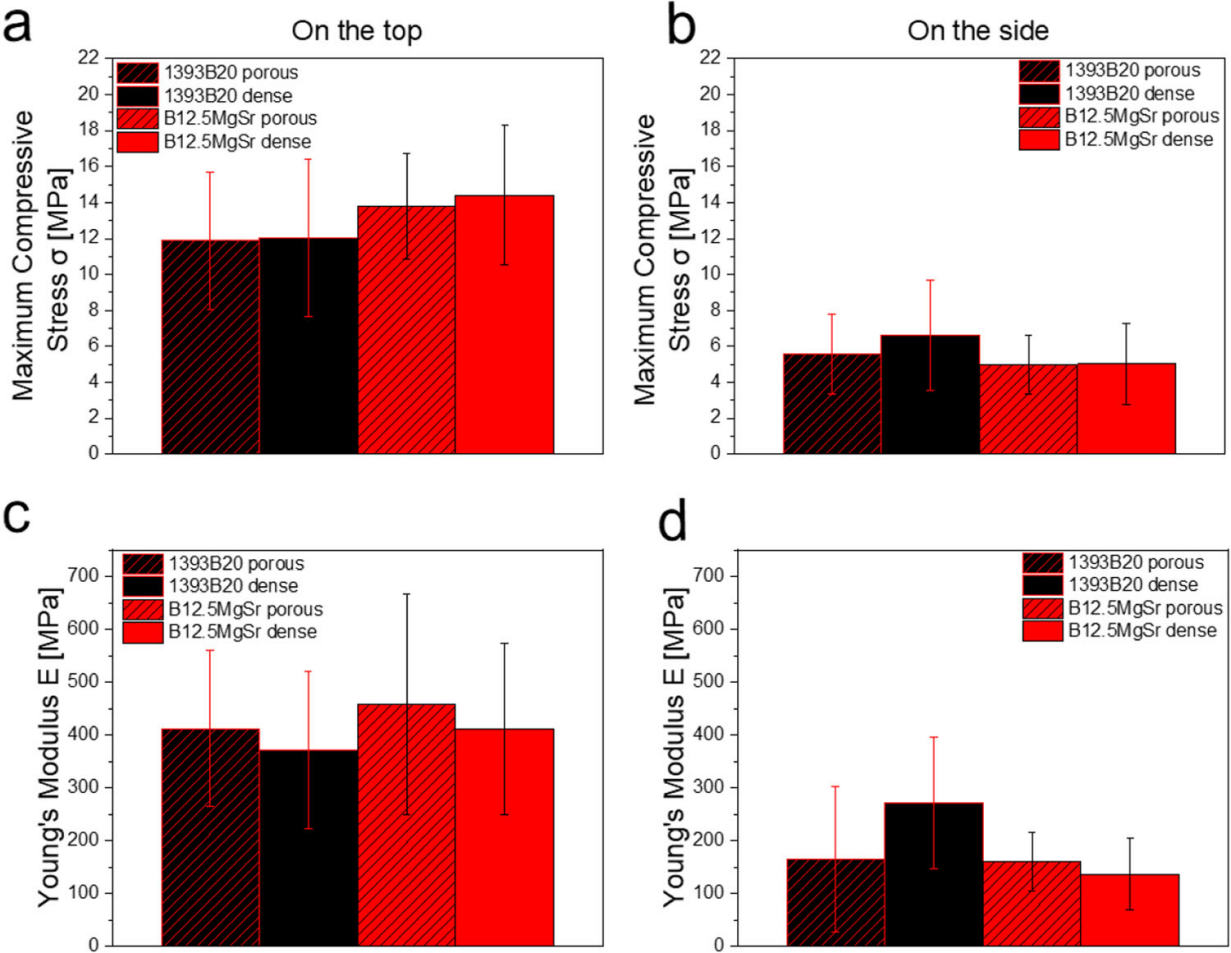
**a**, **b** Maximum compressive stress and **c**, **d** Young’s modulus at failure of B12.5MgSr and 1393B20 scaffolds with and without dense top. Measured by applying force **a**, **c** on the top and **b**, **d** on the side of the scaffolds

The mechanical properties were investigated by evaluating maximum compressive stress (Fig. 3a, b) and Young’s modulus (Fig. 3c, d). Due to the presence of the dense layer, it is expected that the force will distribute differently if applied on the side of the scaffold or on the top of the scaffold. Thus, the measurement was carried out in two modes, with force applied on the top (Fig. 3a, c) and on the side of the scaffolds (Fig. 3b, d).

Despite the different top (porous and dense), in either mode, there is no significant difference in mechanical properties between different scaffold types and glass compositions. The dense layer is relatively thin to have any significant effect on mechanical properties between scaffolds. Moreover, the porosity of the 1393B20 and B12.5MgSr BAG scaffolds are comparable, resulting in similar mechanical properties.

In conclusion, the large porosity and the defects induced during particle sintering are dominating the overall mechanical properties of the materials for all the conditions, as observed by D’Andrea et al. [47].

Further, when scaffolds’ mechanical behaviour, between different modes of the measurement, is compared, significant difference is observed. When the force is applied to the side of the scaffolds, the Young’s modulus and maximum compressive stress values for all scaffolds are 43 and 35% lower than the values measured with force applied to the top of the scaffolds, respectively. It seems that scaffolds withstand the force better when it is applied to the top of the scaffolds, no matter if top is porous or dense. This indicates that some anisotropy in the scaffold was produced. Nevertheless, as mentioned above, the overall porosity and internal defects as a results of particles sintering are driving the overall mechanical properties.

Most importantly, the goal for the scaffold is to mimic the mechanical properties of the natural bone. The maximum compressive stress was measured to be around 6 and 14 MPa, for all scaffolds, when force was applied on the side and top of the scaffolds, respectively. The Young’s modulus was measured to be around 180 and 520 MPa for all scaffolds, when the forces were applied on the side and top of the scaffolds, respectively. Summarizing, 1393B20 and B12.5MgSr scaffolds with and without dense top show compressive strength close to the 2–12 MPa of trabecular bone [48]. It has been reported that hip stems and tibial bones are subjected to 3–11 MPa and 4 MPa stresses, respectively [49, 50]. Therefore, regardless of the direction of the applied force and of the composition and structure, all the analyzed scaffolds possess mechanical properties in lines with those of the cortical bone.

### Static in vitro dissolution in TRIS and bioactivity in SBF

**Fig. 4:**
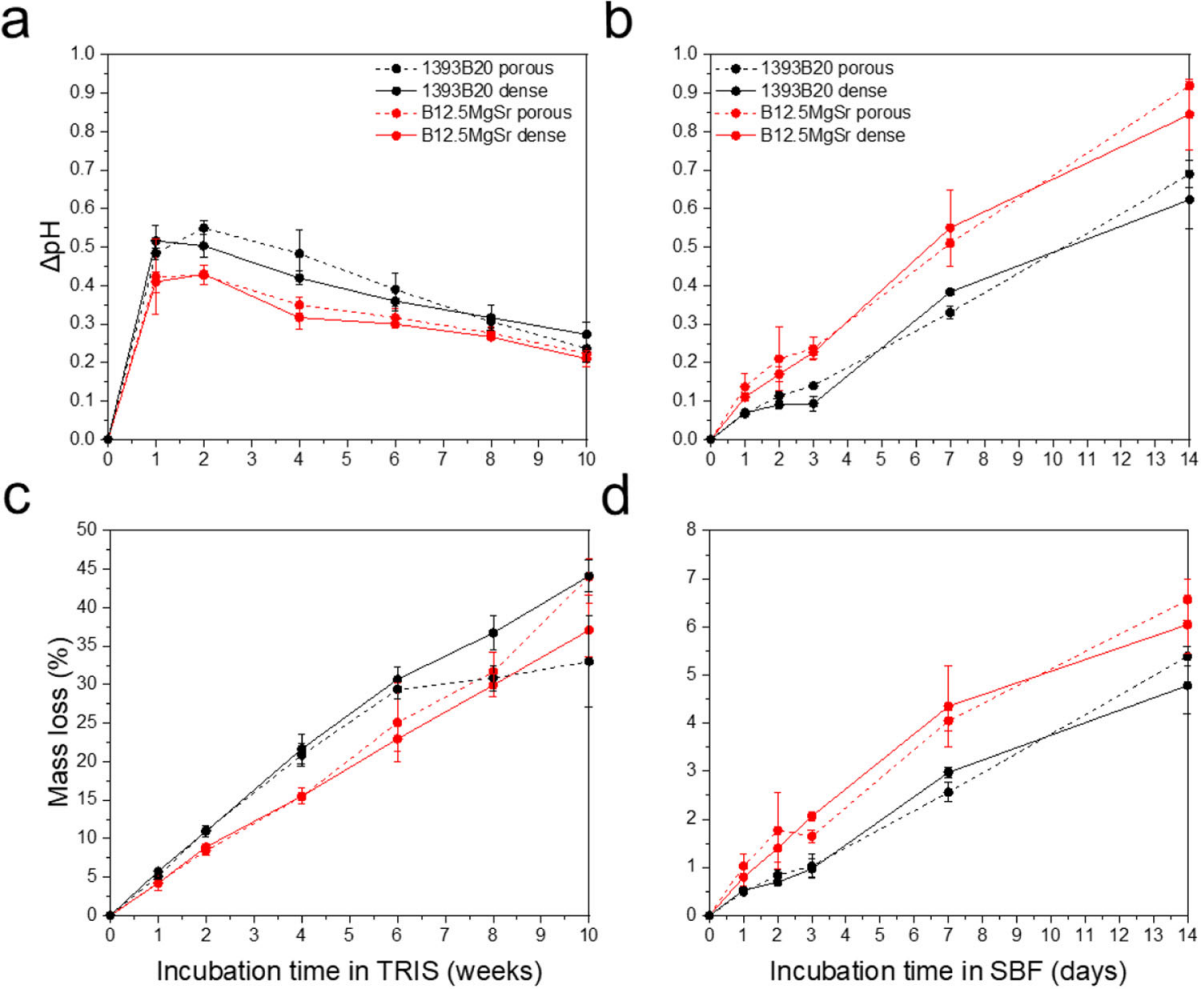
**a**, **b** ΔpH and (**c**, **d**) mass loss after static in vitro dissolution with B12.5MgSr and 1393B20 scaffolds with and without dense top in (**a**, **c**) TRIS (for 10 weeks) and **b**, **d** SBF (for 2 weeks). ΔpH = pH in TRIS/SBF in the presence of the sample – pH in TRIS/SBF control

To study the static in vitro dissolution and bioactivity, the scaffolds were incubated in TRIS for up to 10 weeks and in SBF for up to 2 weeks. In vitro dissolution tests performed in TRIS aimed to assess the ions released from the glass during dissolution over long period of time. TRIS solution was changed every week to avoid saturation of the solution with the leached ions. Dissolution in SBF aimed to assess the ability of the released ions to saturate the solution and to induce the precipitation of a reactive layer. SBF dissolution was done for 2 weeks without refreshing the solution, which is optimal to observe precipitation of HA. The pH variation (ΔpH) of TRIS and SBF uptake solutions as a function of the incubation time for both glass compositions manufactured into 3D printed scaffolds with dense and porous top are presented in Fig. 4.

For all scaffold types and compositions, there is a rise in ΔpH with increasing immersion time followed by stabilization around the week 1 in TRIS (Fig. 4a). However, in SBF the rise in ΔpH was linear during the whole 2 weeks of incubation (Fig. 4b). The initial increase in ΔpH can be associated with the ion release as reported in previous studies [15, 17]. Dissolution of borosilicate bioactive glasses is diffusion controlled and therefore at longer immersion time the speed of release of ions decreases, thus leading to a saturation in the pH increase [51].

In both SBF and TRIS solution, during the first week, ΔpH raised by around 0.4–0.5. After the first week the ΔpH profiles cannot be compared between TRIS and SBF uptake solutions, due to differences in refreshing timepoints. Most importantly, in TRIS, dissolution of 1393B20 scaffolds resulted averagely in higher ΔpH compared to B12.5MgSr, while in SBF the opposite phenomenon was observed. This difference in ion release between scaffolds in SBF and TRIS could be due to different speeds of precipitation of HA. However, no difference was observed in the rise of ΔpH between different scaffold types, with and without dense top.

The ΔpH after 2 weeks of immersion in TRIS (which was refreshed every week) increased up to 0.55 compared to initial pH level of 7.4. In SBF (which was not refreshed over the experiment) pH increased up to 0.92 compared to initial level. This indicates that in both cases the scaffold dissolution is rapid and, if not carefully controlled, may be toxic for cells (Ciraldo et al., 2018).

In the Fig. 4c, d, mass loss during in vitro dissolution in TRIS and SBF is shown. In both immersion solutions the mass loss linearly increases over time. The difference between scaffolds with and without dense top is not significant. Most importantly, in TRIS, 1393B20 scaffolds show higher mass loss, while in SBF B12.5MgSr scaffolds exhibit the highest. This observation is in accordance with ΔpH results. When comparing the mass loss in TRIS and SBF during the first 2 weeks, it can be noticed that mass loss in TRIS is slightly higher (up to 11%) compared to the maximum mass loss after 2 weeks in SBF which is 6.5%.

Summarising, the ΔpH and mass loss results, indicate that dissolution in TRIS is not comparable to dissolution in SBF. B12.5MgSr scaffolds produce higher ΔpH levels in TRIS but lower in SBF compared to 1393B20 scaffolds. Finally, as expected, since their overall porosity being similar 3D printed scaffolds with and without dense top, produce similar ΔpH levels. To further analyse the dissolution behaviour, we looked at ion concentrations after immersion in TRIS and SBF.

**Fig. 5:**
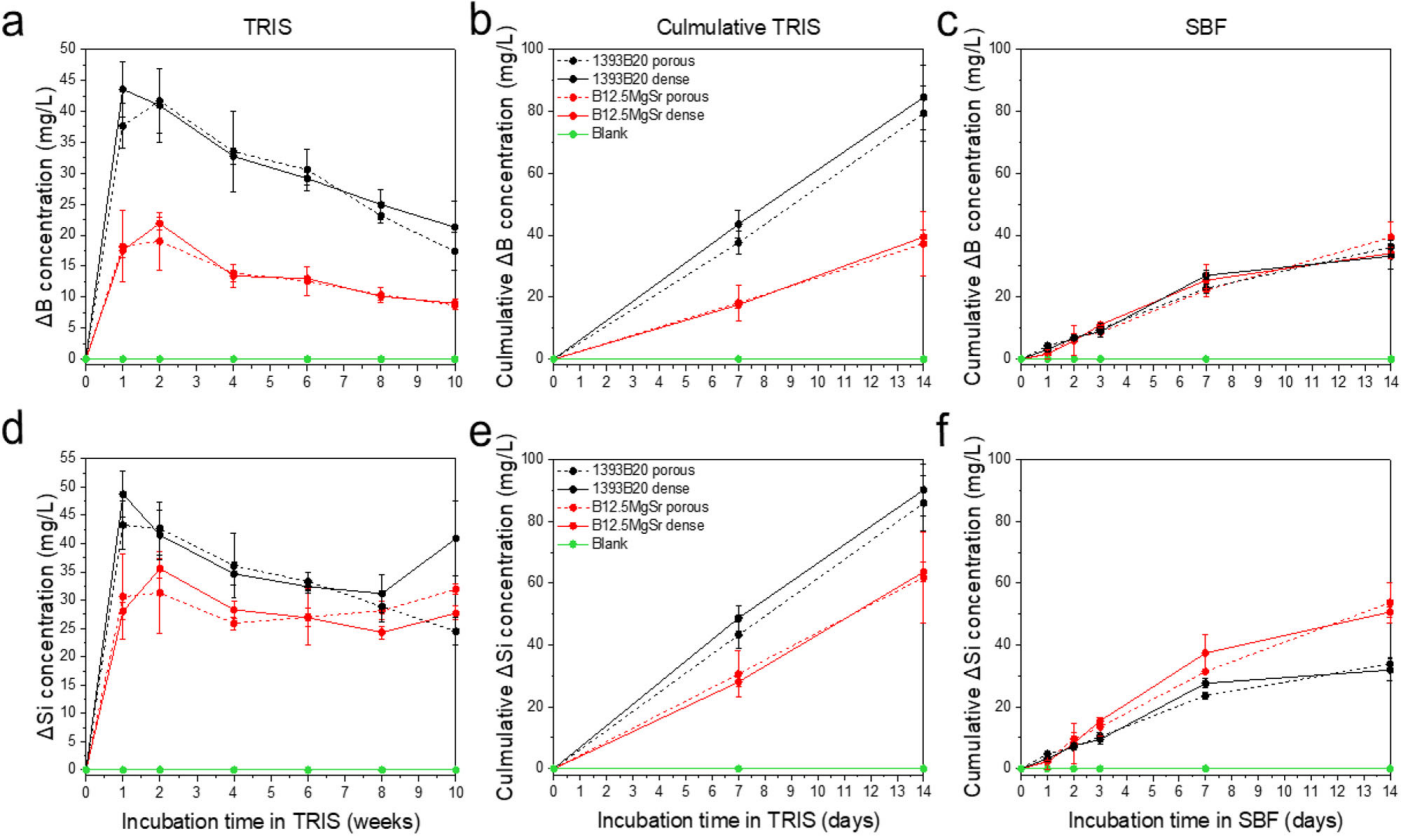
Concentrations of (**a**–**c**) B and (**d**–**f**) Si after static in vitro dissolution with B12.5MgSr and 1393B20 scaffolds with and without dense top in (**a**, **b**, **d**, **e**) TRIS (for 10 weeks) and **c**, **f** SBF (for 2 weeks). ΔElement = [Element] in TRIS/SBF in the presence of the sample – [Element] in TRIS/SBF initial solution

**Fig. 6:**
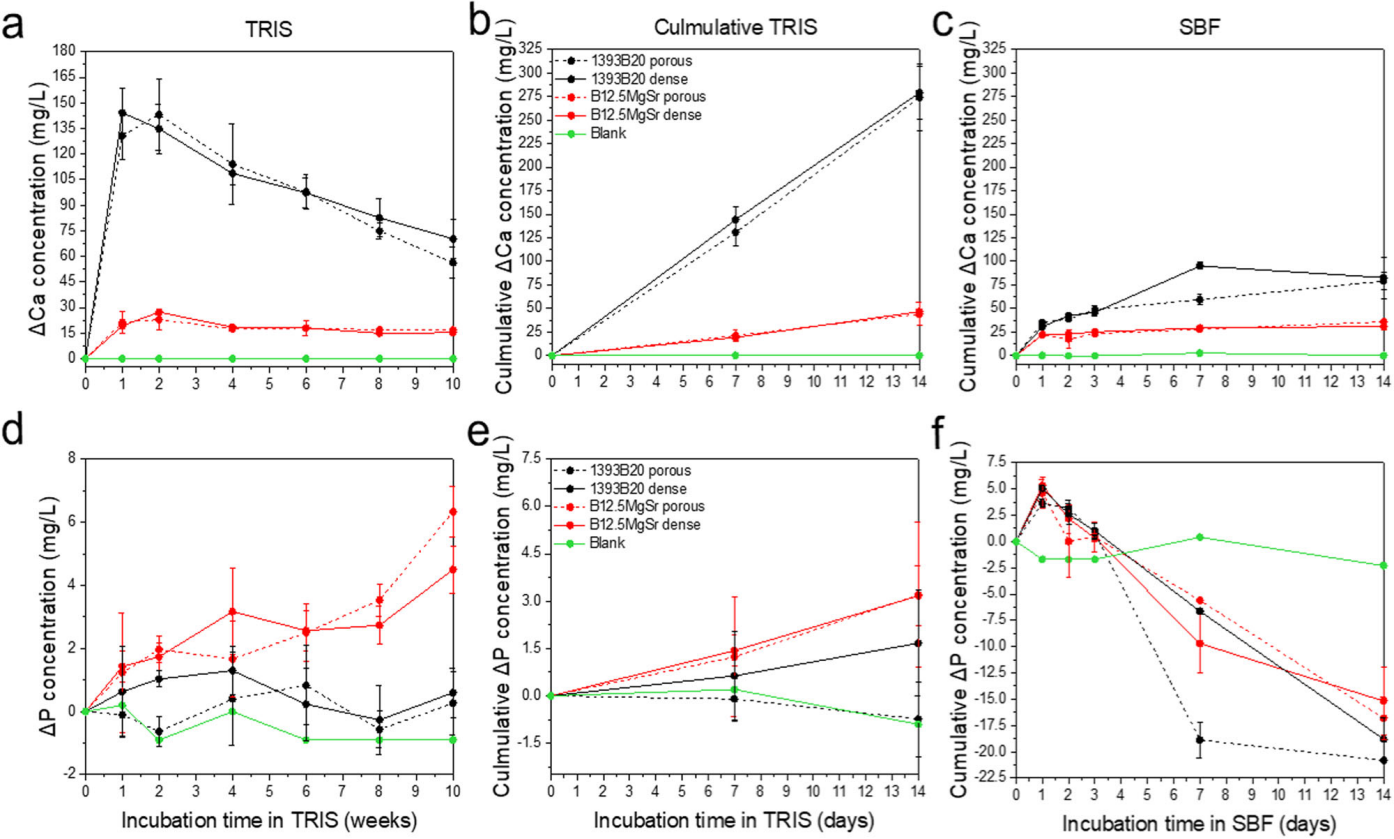
Concentrations of (**a**–**c**) Ca and (**d**–**f**) P after static in vitro dissolution with B12.5MgSr and 1393B20 scaffolds with and without dense top in (**a**, **b**, **d**, **e**) TRIS (for 10 weeks) and (**c**, **f**) SBF (for 2 weeks). ΔElement = [Element] in TRIS/SBF in the presence of the sample – [Element] in TRIS/SBF initial solution

**Fig. 7:**
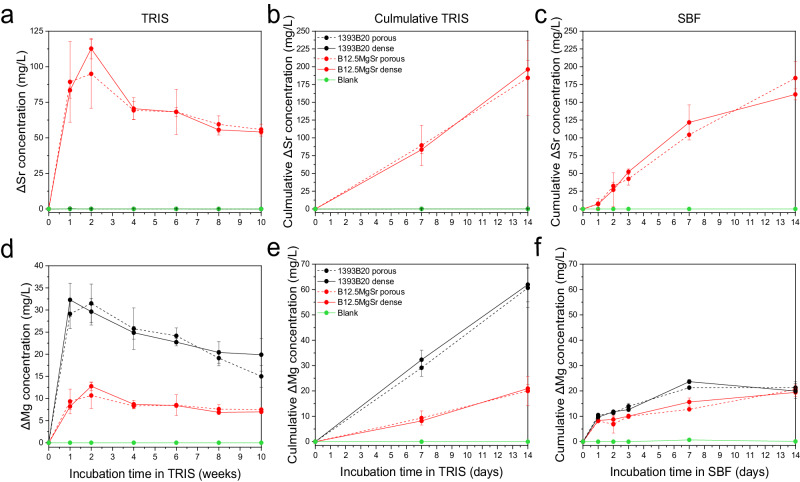
Concentrations of (**a**–**c**) Sr and (**d**–**f**) Mg after static in vitro dissolution with B12.5MgSr and 1393B20 scaffolds with and without dense top in (**a**, **b**, **d**, **e**) TRIS (for 10 weeks) and **c**, **f** SBF (for 2 weeks). ΔElement = [Element] in TRIS/SBF in the presence of the sample – [Element] in TRIS/SBF initial solution

The ion concentrations in TRIS (10 weeks) and SBF (2 weeks) after static in vitro dissolution were analysed using ICP-OES. It is important to note that all graphs that present ion release in SBF show cumulative ion release, because SBF was not refreshed. However, for in vitro dissolution in TRIS ion release was plotted as cumulative and non-cumulative. In non-cumulative graphs (Figs. 5–7a, d), each point gives information about ion release in TRIS during the preceding week. In cumulative graphs (Figs. 5–7b, e) the cumulative release for immersion up to 2 weeks in TRIS is shown, for comparison with the ion release in SBF (Figs. 5–7c, f).

Si and B are backbone of the glass network, and their release gives information about the overall glass dissolution profiles. Si^4+^ and B^3+^ ion release in TRIS and SBF is shown in Fig. 5. In TRIS after the initial fast release of Si^4+^ and B^3+^ ions, dissolution slows down after 1–2 weeks (Fig. 5a, d). The release is larger and faster for 1393B20 compared to B12.5MgSr scaffolds. This could be explained by the fact that 1393B20 has higher B content in its glass composition. Although, 1393B20 scaffolds also release higher levels of Si in TRIS, despite having less Si in its glass composition compared to B12.5MgSr scaffolds.

In SBF the Si^4+^ and B^3+^ cumulative ion releases initially are linear and slow down after one week (Fig. 5c, f). Cumulative release of B is not significantly different between 1393B20 and B12.5MgSr glass composition. Cumulative release of Si in SBF is higher for B12.5MgSr scaffolds.

Overall, cumulative release of Si^4+^ and B^3+^ ions in TRIS is higher than in SBF during the first 2 weeks of immersion (Fig. 5b, c, e, f). Cumulative release in SBF also stabilizes faster. These observations could be explained by an overall faster precipitation of a reactive layer in SBF. Summarizing, 1393B20 scaffolds dissolve faster in TRIS. However, in SBF, dissolution of 1393B20 scaffolds probably leads to faster precipitation, compared to B12.5MgSr glass composition. This causes the differences between ion release profiles in TRIS and SBF. Finally, there is no significant difference between ion release between scaffolds with and without dense top in either TRIS or SBF.

Ca and P release profile gives information about precipitation of CaP layer on the surface of scaffolds and, thus, scaffolds bioactivity (Fig. 6).

Ca release from B12.5MgSr scaffolds is overall stable during the whole time of the experiment in TRIS (Fig. 6a). Ca release from 1393B20 scaffolds in TRIS is characterized by initial fast release of ions followed by slow release after 1–2 weeks. Cumulative Ca release in SBF is initially linear and slows down after week 1. Over all, cumulative Ca^2+^ ion release in SBF, stabilizes faster than in TRIS (Fig. 6b, c). This can again be explained by probably faster precipitation in SBF [52]. Moreover, the cumulative release of Ca^2+^ ions in both SBF and TRIS is higher from 1393B20 scaffolds. As expected, since 1393B20 scaffolds have three times higher Ca content in its glass composition.

P release from scaffolds in TRIS does not follow any specific release profile, which could be explained by simultaneous release and precipitation of P (Fig. 6d). Moreover, P release in TRIS is more significant from B12.5MgSr compared to 1393B20 scaffolds.

As expected, cumulative ion release in SBF indicates precipitation of P for both 1393B20 and B12.5MgSr scaffolds already after 1 day of immersion (Fig. 6f). Consumption of P indicates precipitation of CaP layer indicative of scaffolds bioactivity [53]. This precipitation is not significantly faster for any glass composition or scaffold type. As expected, since TRIS solution was refreshed, the cumulative release of P in TRIS is linear and does not show precipitation (Fig. 6e).

Summarising, cumulative release of Ca^2+^ and P^3-^ ions in SBF indicate precipitation of CaP like layer and scaffolds bioactivity. Finally, there is no significant difference between Ca^2+^ and P^3-^ ion releases between scaffolds with and without dense top in either TRIS or SBF.

Sr^2+^ ion release profiles are shown in the Fig. 7a–c. 1393B20 scaffolds are the only one that contains Sr in their glass composition. Sr^2+^ ion release profile in TRIS is characterized by initial fast release that reaches maximum at week 2, followed by slow release (Fig. 7a). Cumulative Sr release during first 2 weeks in TRIS and in SBF is linear and reach similar concentration levels (Fig. 7b, c). There is no significant difference between scaffolds with and without dense top.

Analogously, Mg^2+^ ion release profile in TRIS is characterised by initial fast release and followed by slow release, especially with 1393B20 scaffolds (Fig. 7d). Mg content in 1393B20 glass composition is 1.5 times higher compared to B12.5MgSr glass composition. Thus, as expected, 1393B20 scaffolds release more Mg, both in TRIS and in SBF. However, this difference in Mg^2+^ ion release between different glass compositions is significantly larger in TRIS than in SBF. Moreover, the cumulative Mg^2+^ ion release during first 2 weeks of immersion is significantly higher in TRIS compared to SBF (Fig. 7e, f). This phenomenon could be due to the presence of Mg in SBF, which slows down the Mg release and the diffusion process. The cumulative release profile of Mg in TRIS during the first 2 weeks of immersion is linear, while in SBF the cumulative release of Mg stabilizes after 1 day, significantly earlier than in TRIS, again probably due to faster HA precipitation in SBF.

**Fig. 8:**
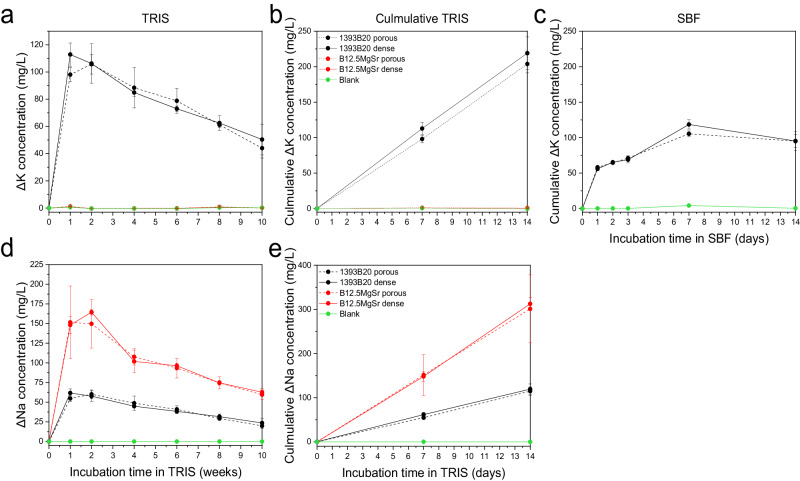
Concentrations of (**a**–**c**) K and (**d**, **e**) Na after static in vitro dissolution with B12.5MgSr and 1393B20 scaffolds with and without dense top in (**a**, **b**, **d**, **e**) TRIS (for 10 weeks) and (**c**) SBF (for 2 weeks). Na concentration in SBF is not reported because oversaturated. ΔElement = [Element] in TRIS/SBF in the presence of the sample – [Element] in TRIS/SBF initial solution

Release of K and Na is shown in the Fig. 8a–c. Only 1393B20 glass composition contains K. K released in TRIS is characterized by initial fast release, reaching the maximum at 1–2 weeks, followed by slow release (Fig. 8a). K cumulative release in TRIS during the first 2 weeks follows a linear profile and is more profound compared to release in SBF (Fig. 8b, c). K cumulative release in SBF slows down after 1 day but overall does not show any characteristic profile (Fig. 8c). There is no difference between scaffolds with and without dense top.

Finally, Na release profiles could be recorded only in TRIS (Fig. 8d, e). In SBF, there is oversaturation of Na^+^ ions that makes it impossible to quantify them correctly. Na release profile in TRIS is characterised by initial burst release followed by a s after 1–2 weeks. Cumulative release of Na in TRIS during first 2 weeks is linear (Fig. 8e). B12.5MgSr shows higher Na^+^ ions release in TRIS compared to 1393B20 glass composition. This can be explained by almost 4 times higher content of Na in B12.5MgSr glass composition.

Overall, the ion dissolution results in TRIS and SBF, confirm the significant differences in release profiles between TRIS and SBF previously hypothesized from ΔpH results. Moreover, Ca stabilization and P precipitation in SBF are indicative of scaffolds bioactivity. This further confirms that most probably the differences in ion release profiles and ΔpH results are due to different rates of precipitation of CaP HA-like layer on the surface of the scaffold immersed in SBF and TRIS. In vitro dissolution in TRIS and SBF is sometimes used to predict the performance of scaffolds in culture with cells or in vivo. However, due to differences between ion release in TRIS and SBF, these results should be extrapolated cautiously to estimate ion release profiles in culture medium.

### Structural properties – FTIR-ATR spectroscopy

**Fig. 9:**
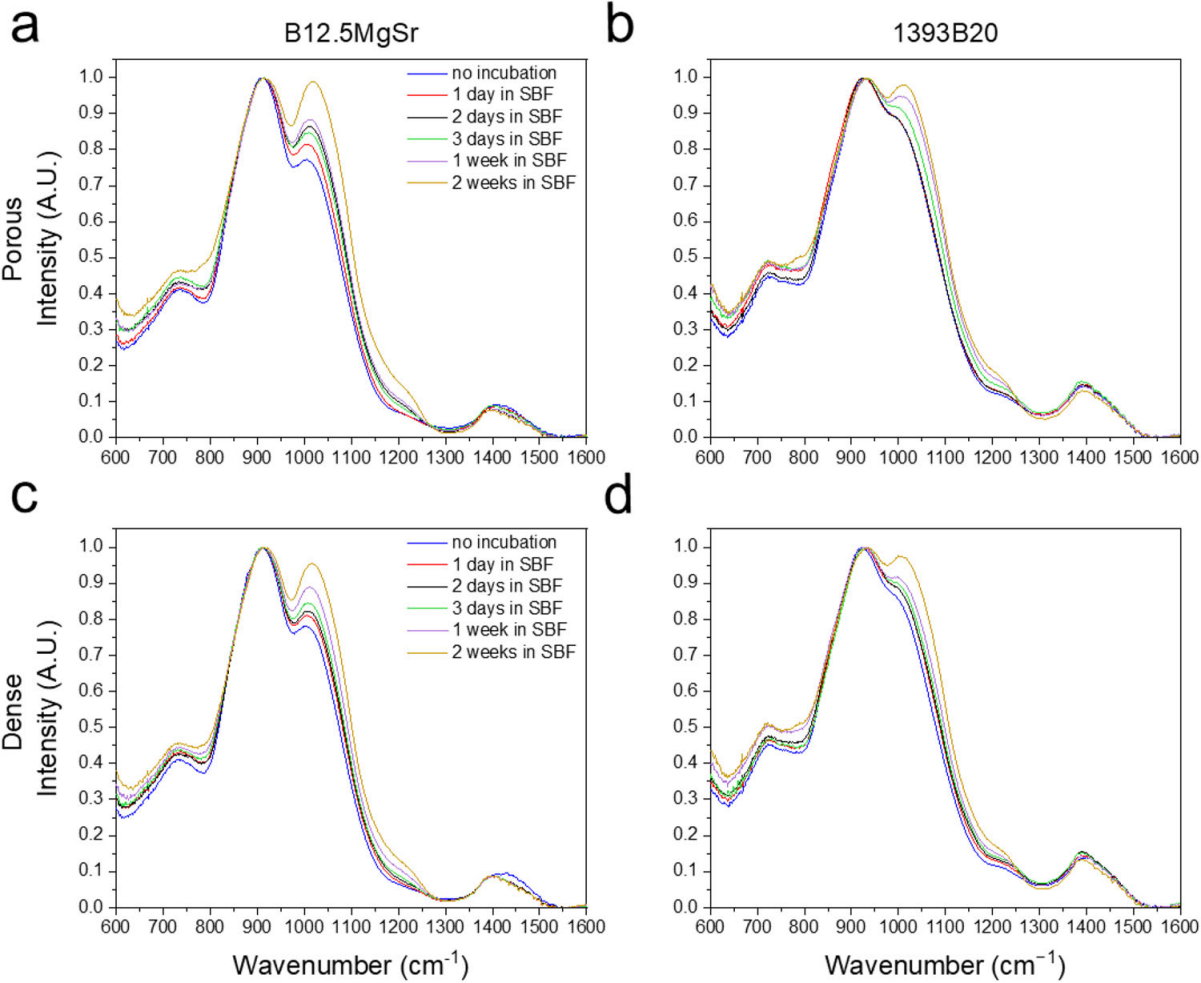
FTIR-ATR spectra of the BAG scaffolds before and after 2 weeks immersion in SBF: (**a**, **c**) B12.5MgSr scaffolds, (**b**, **d**) 1393B20 scaffolds with (**a**, **b**) porous top and (**c**, **d**) dense top

Changes in glass surface composition before and during the 2 weeks immersion in SBF were assessed using FTIR-ATR spectroscopy (Fig. 9). The spectra were acquired at each time point.

Spectra for all glass compositions and scaffold types show the same peaks, which are characteristic of borosilicate glasses. The band at ~730 cm^−1^ detected on both 1393B20 and B12.5MgSr before the immersion can be assigned to Si-O bending [16, 17]. The high-intensity band at ~1010 cm^−1^ corresponds to Si-O-Si asymmetric stretching as well as to B-O stretching vibration in [BO_4_] units [54–56]. The low intensity band at ~1227 cm^−1^ can be associated with vibrations of [BO_2_O^-^] units and increases with immersion time [5, 56]. Finally, the lower intensity band at ~1400 cm^−1^ can be attributed to B-O stretching vibration in [BO_3_] units [57].

It is challenging to precisely analyze and compare FTIR-ATR spectra between 1393B20 and B12.5MgSr bioactive glass due to the overlapping of silica and borate related peaks. Nevertheless, the FTIR-ATR spectra of these glasses are similar, which may indicate that they have similar network connectivity.

With increasing immersion time for both B12.5MgSr and 1393B20 BAG scaffolds with and without dense top, 1) the band located at ~730 cm^−1^ increased in intensity, 2) the band located at ~1010 cm^−1^ increased in intensity and shifted to higher wavenumbers, 3) the low intensity band at ~1227 cm^−1^ increased in intensity, and 4) the lower intensity band at ~1400 cm^−1^ decreased in intensity and shifts to lower wavenumber.

The bands at ~1010 cm^−1^ and ~910 cm^−1^ after immersion can be attributed to P-O and P = O stretching vibrations, respectively. Moreover, after immersion, the low intensity band at ~1227 cm^−1^ can be also explained by Si [Q_4_] units and presence of the silica gel. [16, 58, 59]

Moreover, a slight decrease in the intensity of the band at ~1400 cm^−1^ could indicate that boron structure is impacted by immersion, probably by dissolution of borate network [57]. Finally, the appearance of the band at 3000–3550 cm^−1^ (not shown) which increases in intensity after immersion suggest presence of hydroxyl or silanol groups implying presence of water [54, 59].

Furthermore, in case of B12.5MgSr, after immersion, the band at ~1010 cm^−1^ changed more significantly compared to 1393B20 glass composition. This indicate a faster reactivity of the B12.5MgSr compared to 1393B20 [5, 16]. This is in accordance with ΔpH results during dissolution in SBF also indicating faster dissolution of B12.5MgSr scaffolds.

Summarizing, the FTIR-ATR spectroscopy results, before and after 2 weeks immersion in SBF, suggest that scaffolds dissolves which results in precipitation of calcium phosphate surface layer. This dissolution is suggested to be faster in the case of B12.5MgSr.

### Mean pore size and porosity pre- and post-immersion in SBF

**Fig. 10:**
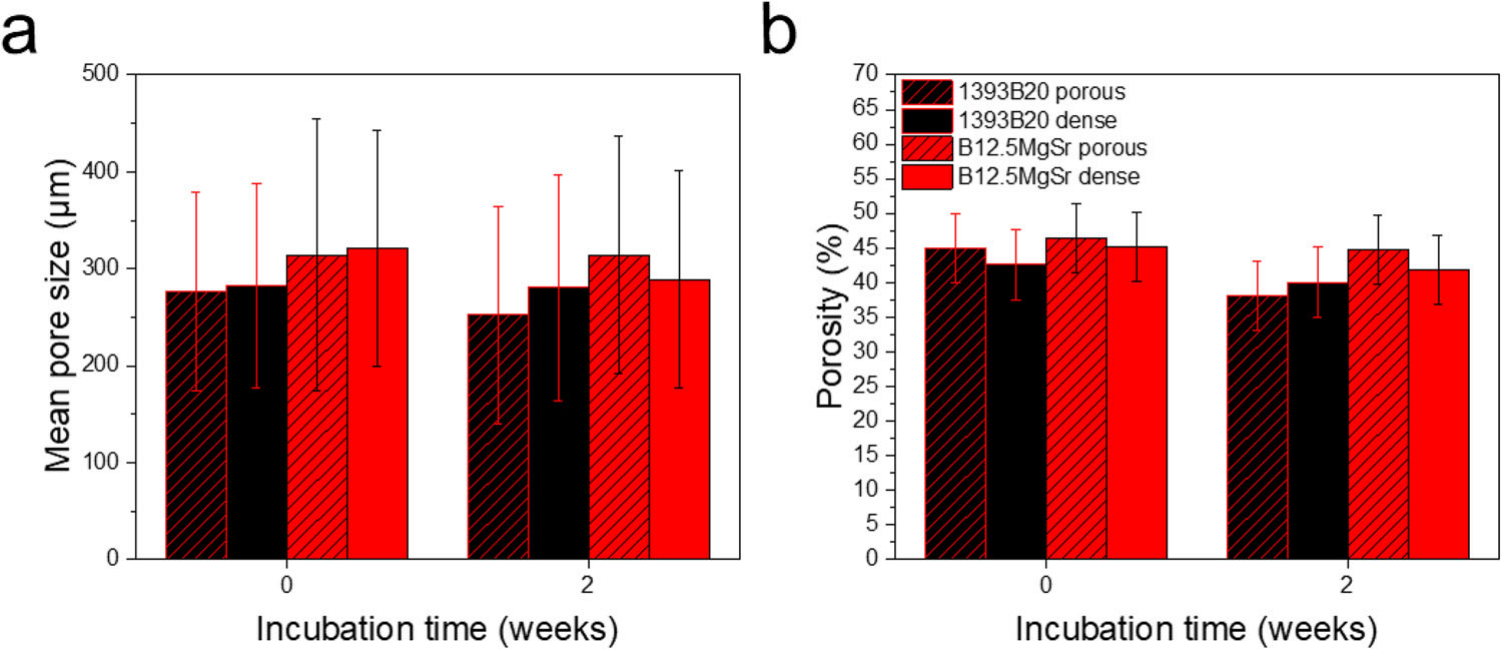
**a** Mean pore size and (**b**) porosity of B12.5MgSr and 1393B20 scaffolds with and without dense top

From the µCT images of the scaffold acquired before and after 2 weeks immersion in SBF, the mean pore size and the overall porosity were calculated. The results are presented in Fig. 10.

Immersion of scaffolds in SBF had no significant effect on the mean pore size and porosity of the B12.5MgSr and 1393B20 scaffolds with and without dense top.

### Surface analysis

**Fig. 11:**
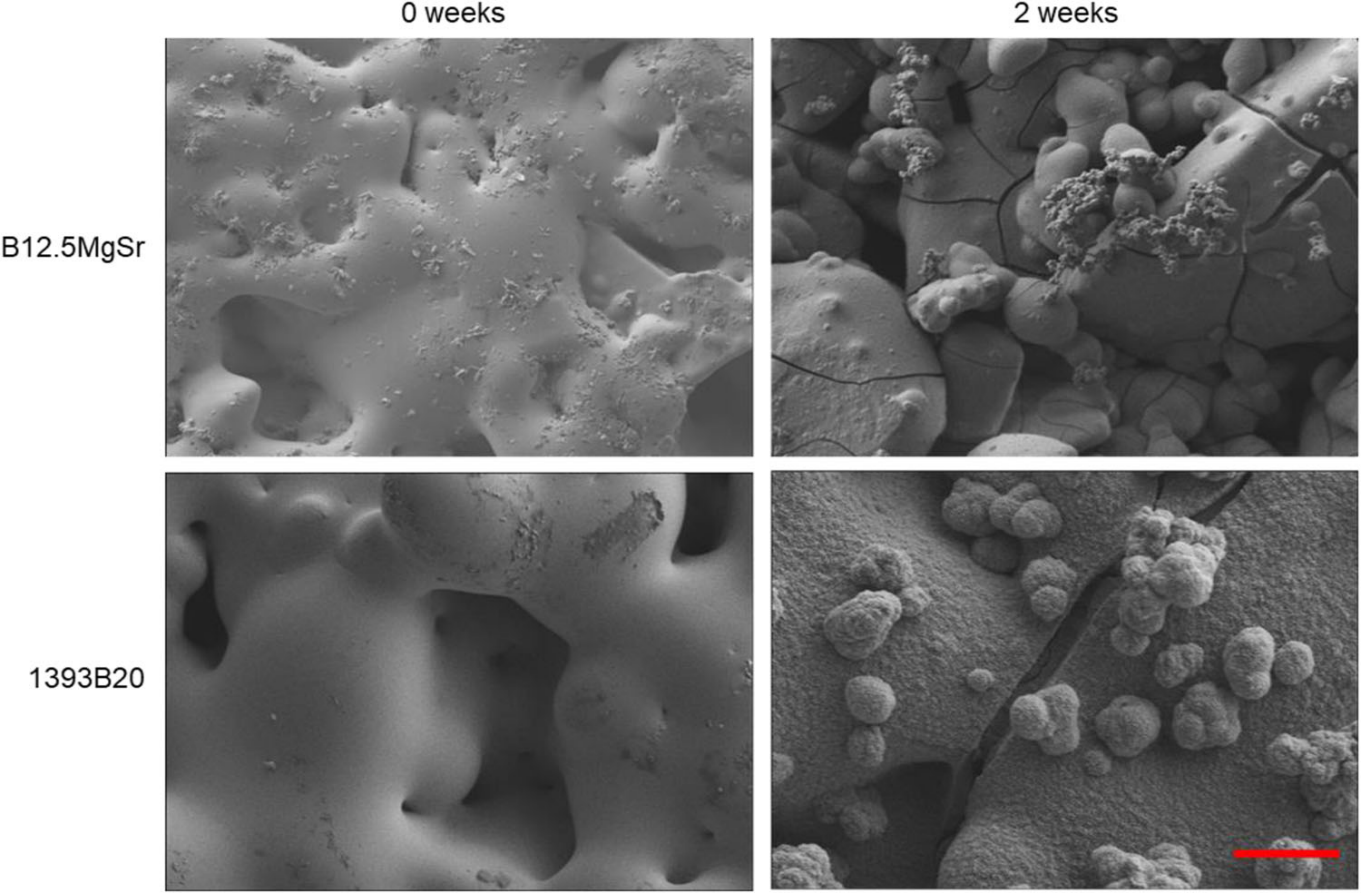
SEM images acquired before and after the immersion in SBF for 2 weeks. Scale bar 10 µm. The image is representative for both B12.5MgSr and 1393B20 glasses

SEM-EDX was performed to further investigate the precipitation post-immersion in SBF. SEM images taken at the scaffolds top surface before and after 2 weeks incubation in SBF are shown in Fig. 11.

After 2 weeks incubation in SBF, nodules appeared at the surface of B12.5MgSr and 1393B20 scaffolds. At the surface of 1393B20 nodules are averagely bigger and reach ~5 µm in size. Cracks can be also visible in the precipitation layer. Moreover, the EDX analysis showed that the composition of the nodules at the surface of 1393B20 scaffolds led to the characteristic ratio (Ca+Mg)/P ~ 1.79 ± 0.28 which is close to 1.67, typical of HA [60]. Precipitates at the surface of B12.5MgSr scaffolds exhibited (Ca+Sr+Mg)/P ratio ~ 1.96 ± 0.32. This ratio although higher than previously reported for B12.5MgSr, is still close to HA [15]. Summarizing, together with ion release profiles in SBF and FTIR-ATR results, SEM images confirm that all scaffold types precipitated calcium phosphate reactive layer with a composition close to HA, which is one of the first sights of bioactivity. Moreover, Sr and Mg are also incorporated into the precipitated CaP reactive layer, which is in accordance with previous results [15–17].

### Effect of preincubation on burst release of ions

So far, it has been shown that there was no significant difference in ion dissolution, porosity, mechanical properties, and structural properties between scaffolds with dense and porous top. However, scaffolds with dense top exhibit gradient of porosity more closely mimicking the natural bone. Furthermore, dense layer at the top of the scaffold allows for potential membrane deposition, often used in bone guiding regeneration and, as such, opens the path to multifunctional scaffold, not only able to repair bone, but also able to prevent soft tissue infiltration [36]. Thus, for preincubation and cell culture experiments, only 3D printed scaffolds with dense top were used.

**Fig. 12:**
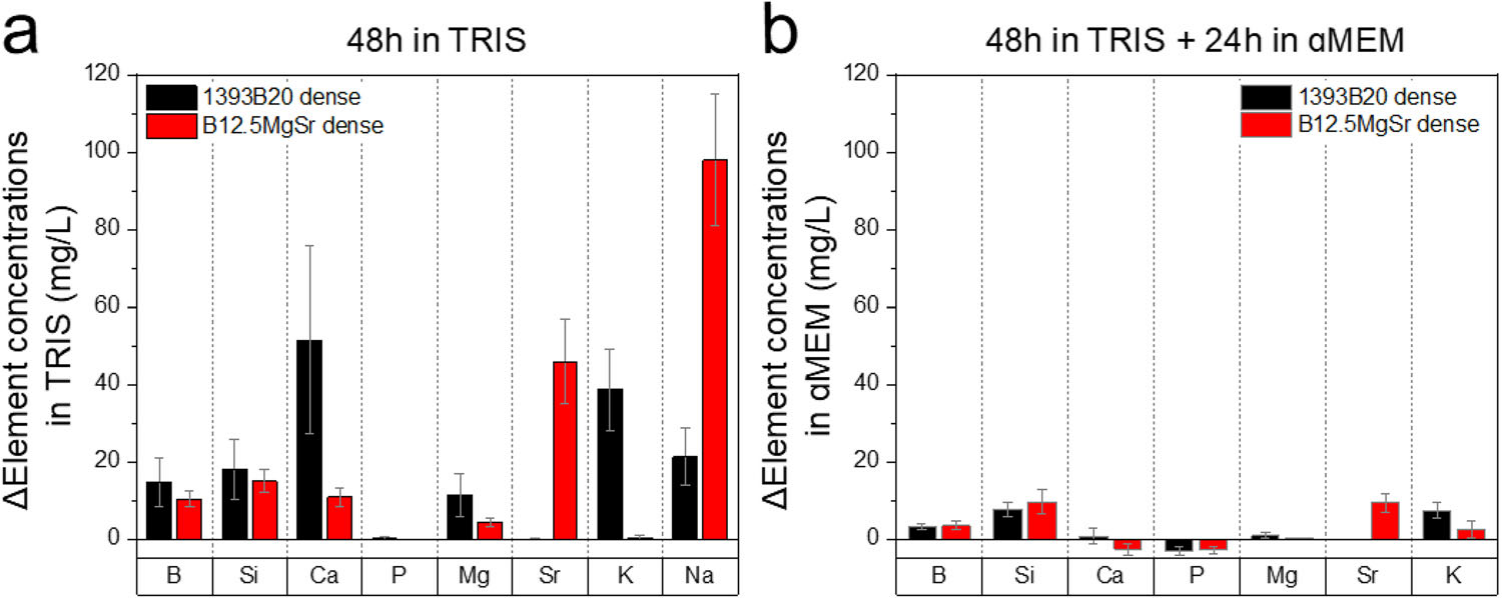
Concentrations of B, Si, Ca, P, Mg, Sr, K and Na after preincubation of 3D printed B12.5MgSr and 1393B20 scaffolds with dense top for 48 h in TRIS and subsequent 24 h in αMEM. ΔElement = [Element] in TRIS/αMEM in the presence of the sample – [Element] in TRIS/αMEM initial solution

To assess the amount of ions released during the preincubation, scaffolds were immersed according to previously optimized protocol: 48 h in TRIS and subsequently for 24 h in αMEM [35]. After each step of preincubation ions concentrations were measured using ICP-OES (Fig. 12).

After 48 h preincubation in TRIS, Si^4+^ and B^3+^ ion release was higher for 1393B20 (Fig. 12a). After additional 24 h preincubation in αMEM, the release of B and Si decreased. Moreover, after 24 h in αMEM there was no significant difference in B and Si release between 1393B20 and B12.5MgSr scaffolds (Fig. 12b).

Ca^2+^ ion release, after 48 h preincubation in TRIS, was higher from 1393B20 scaffolds (Fig. 12a). P release in TRIS seemed close to zero for both scaffold types (Fig. 12a). However, after further 24 h in αMEM, we can see that both Ca and P show negative values indicating precipitation of CaP layer (Fig. 12b). The difference in precipitation is not significant between different glass compositions.

Mg^2+^ ion release after preincubation in TRIS shows higher release from 1393B20 scaffolds compared to B12.5MgSr ones (Fig. 12a). In both glass compositions the Mg release is close to 0 after 3 days of total preincubation time (Fig. 12b).

Sr, present only in B12.5MgSr glass composition, showed a significantly lower ion release after 3 days of total preincubation time.

K, present only in 1393B20 glass composition, significantly decreased after 3 days of total preincubation time.

Na release could be measured only in TRIS, due to Na oversaturation in αMEM (Fig. 12a). Na release is higher from B12.5MgSr compared to 1393B20 scaffolds.

Overall, release of ions during preincubation in TRIS, is in accordance with results of in vitro dissolution in TRIS, during which 1393B20 scaffolds released more ions compared to B12.5MgSr scaffolds. Moreover, the preincubation results shown that 3 days of total preincubation time (48 h in TRIS + 24 h in αMEM) significantly decrease the release of ions. Consequently, preincubation before cell culture experiments can effectively decrease initial burst release of ions that can be cytotoxic.

### Effect of glass composition on cell viability, proliferation, and morphology

The impact of the glass composition on hADSCs survival, proliferation and morphology was investigated in direct culture with 3D printed scaffolds with dense top, post-preincubation for 48 h in TRIS and 24 h in αMEM.

#### Cell viability

**Fig. 13:**
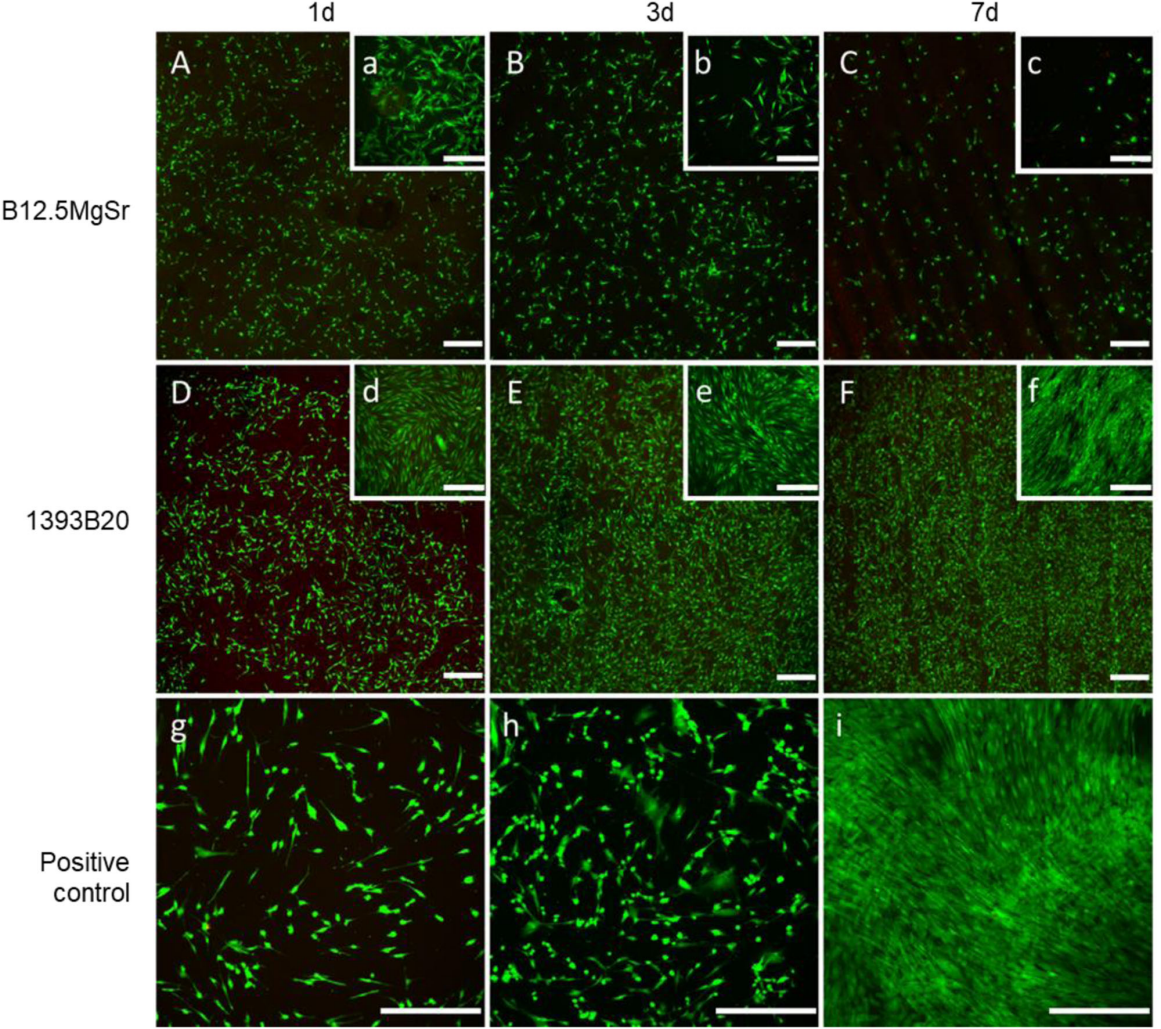
Cell viability of hADSCs after 1, 3 and 7 days of culture in αMEM culture medium. Fluorescence microscope images A-F show the dense top of scaffolds. Images a-i show the bottom of the wells. Viable (green) and necrotic (red) cells were stained with Calcein AM and Ethidium homodimer-1 respectively. Scale bar 400 µm

Fluorescence microscope images of hADSCs after 1, 3 and 7 days of culture with 1393B20 and B12.5MgSr scaffolds with top dense layer are shown in Fig. 13. Cells cultured with 1393B20 scaffolds show better cell viability both at the bottom of the well and at the top of the scaffolds compared to the cells cultured on B12.5MgSr scaffolds. Cells cultured with 1393B20 scaffolds seem to reach confluence around day 3. The viability and cell density of cells cultured with B12.5MgSr scaffolds decreased over time both on the top of the scaffolds and on the bottom of the well. Cell survival around 1393B20 scaffolds is comparable to positive control as both are 2D culture. However, cells on top of the scaffolds are consider as 3D culture, thus, their comparison to positive control is not straight forward.

**Fig. 14:**
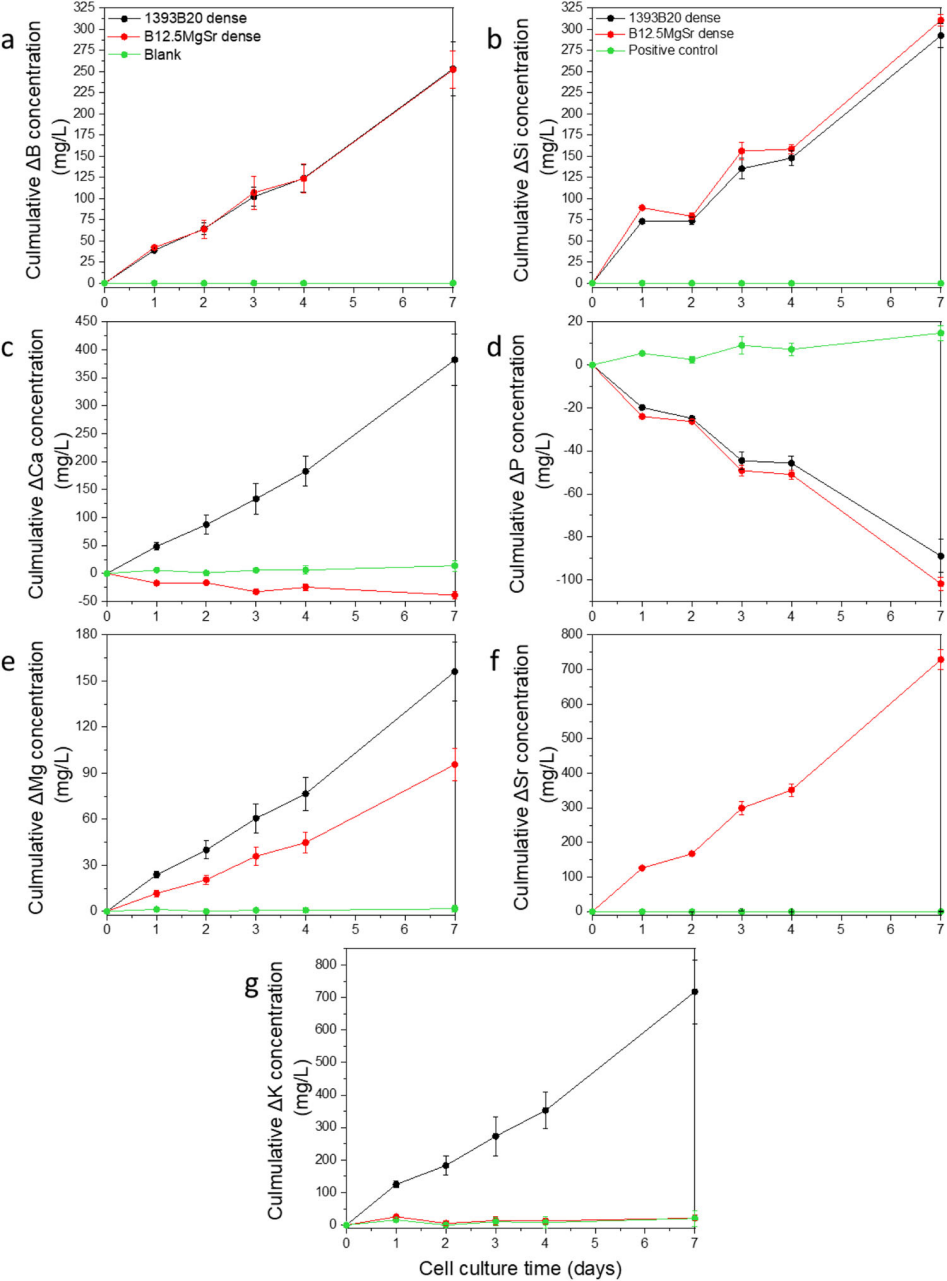
Concentrations of (**a**–**g**) B, Si, Ca, P, Mg, Sr and K in αMEM culture medium after up to 7 days of hADSCs culture with 3D printed B12.5MgSr and 1393B20 scaffolds with dense top as a function of time. ΔElement = [Element] in αMEM in the presence of the sample – [Element] in αMEM initial solution

To understand why 1393B20 scaffolds allow better cell survival and proliferation compared to B12.5MgSr scaffolds, the ion concentrations in αMEM cell culture medium were analysed using ICP-OES (Fig. 14).

The Si^4+^, B^3+^, Mg^2+^ ions release into culture medium is linear (Fig. 14a, b, e). The P^3-^ ion release into culture medium is decreasing linearly indicating CaP precipitation (Fig. 14d). However, the differences in these ion release does not seem significant enough to explain difference in cell viability between different glass compositions. This indicates that difference in hADSCs viability between 1393B20 and B12.5MgSr scaffolds is not due to release of Si, B, Mg and P into the medium.

Sr is found only in B12.5MgSr glass composition (Fig. 14f). Thus, the Sr^2+^ ion linear release was observed only from B12.5MgSr scaffolds, till final cumulative levels of ~729 mg/L. At any given timepoint, the Sr release was not higher than 210 mg/L. In the study of Gentlemen et al., Sr concentrations between 5 and 23 mg/L resulted in enhanced Saos-2 osteoblast cells activity and inhibited osteoclasts differentiation [61]. Moreover, in our previous work, hADSCs cell viability was better with B12.5MgSr scaffolds compared to scaffolds without Sr and Mg (B12.5) [35]. In that study, non-cumulative Sr release at any given timepoint from 3D printed B12.5MgSr scaffolds was maximum 333 mg/L and did not show cytotoxicity. Thus, release of Sr from B12.5MgSr scaffolds is not likely to be the reason for the enhanced hADSCs viability with 1393B20 scaffolds.

K is part only of 1393B20 glass composition (Fig. 14g). Thus, linear release of K^+^ ions is observed only for 1393B20 scaffolds. Concentration of K in culture medium with B12.5MgSr is the same as in blank control. Thus, difference in K release between glass compositions should not be a reason for decreased hADSCs viability with B12.5MgSr.

The concentration of Na is not reported here as the high initial concentration of sodium in αMEM led to oversaturation and consequently inaccurate measurement.

Summarizing, the B^3+^, Si^4+^, P^3-^ and Mg^2+^ ion release were not significantly different between B12.5MgSr and 1393B20 glass scaffolds to explain the variation in hADSCs viability. Strontium release at the same concentrations observed in our previous study was shown not to be cytotoxic [35]. Moreover, Sr release was shown to be beneficial for viability and proliferation of human gingival fibroblasts and osteosarcoma cells [61, 62]. Also, addition of Sr has been shown to promote the proliferation and differentiation of osteoblasts [23–25] and to stimulate an osteogenic response from hBMSCs [26, 27]. Thus, the authors believe that Sr release from B12.5MgSr should have beneficial effect on cell survival.

Ca release profile from 1393B20 scaffolds is linear in culture medium (Fig. 14c). However, when cells are cultured with B12.5MgSr scaffolds, Ca depletion is observed. This indicates that the reactive layer precipitation seems more significant with B12.5MgSr glass scaffolds.

Moreover, during the first 3 days of cell culture with B12.5MgSr scaffolds, the Ca content was on average almost 24 ± 0.8% lower than in culture medium blank. Calcium in culture medium facilitates cells attachment and affects cell movement and shape. Inappropriate calcium amount in culture medium may affect differentiation and viability of cells [63–66]. Thus, Ca depletion from cell culture could be a reason for the slower proliferation and cell viability when hADSCs are cultured with B12.5MgSr scaffolds.

Furthermore, ion release profiles in culture medium are coherent with profiles from the bioactivity test in SBF more closely than from in vitro dissolution in TRIS. Just like in SBF, during cell culture in αMEM, 1) higher contents of Si were released from B12.5MgSr scaffolds compared to 1393B20 scaffolds, 2) B^3+^ ion release was comparable between 1393B20 and B12.5MgSr, 3) fast precipitation of P was observed. This could indicate that release of ions in αMEM, resembles release in SBF, which allows to compare the occurring phenomena. As it was observed during in vitro dissolution tests, B12.5MgSr scaffolds produced higher pH levels, when immersed in SBF, than 1393B20 scaffolds. Thus, cell culture in αMEM with B12.5MgSr scaffolds could result in higher pH levels, compared to culture with 1393B20 scaffolds. Higher pH and depletion of Ca when cells were cultured with B12.5MgSr scaffolds could have led to decrease cell viability.

#### Cell proliferation

**Fig. 15:**
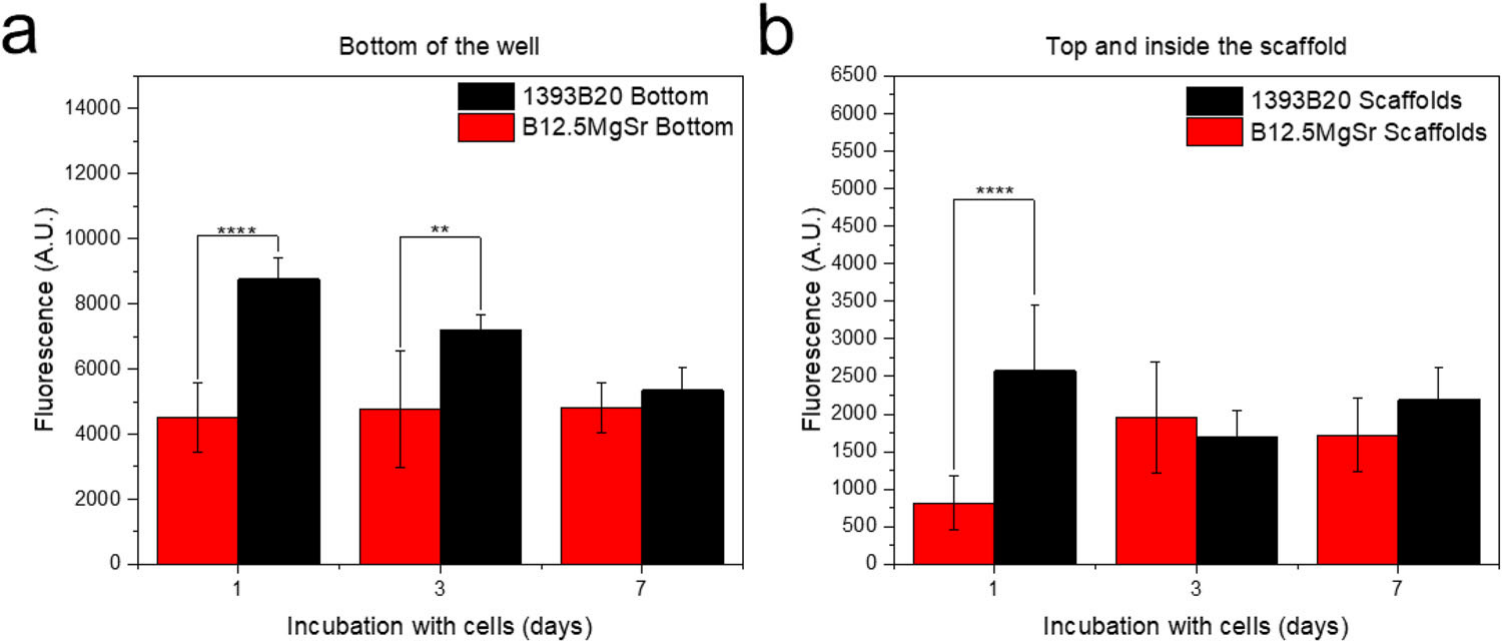
Proliferation of hADSCs cultured with B12.5MgSr and 1393B20 scaffolds in αMEM for 1, 3 and 7 days, (**a**) at the bottom of the wells and (**b**) at the top and in the inside of the scaffolds (*p < 0.005, **p < 0.01, ***p < 0.001, ****p < 0.0001)

Cell proliferation was studied thanks to the CyQUANT^TM^ cell proliferation assay. Cells were quantified at the bottom of the wells (Fig. 15a) and in contact with the scaffolds (Fig. 15b). At day 1 and 3, cells at the bottom of the wells containing 1393B20 scaffolds showed significantly higher cell amounts compared to B12.5MgSr. At D7, the difference between scaffolds is not significant. Moreover, at the bottom of the well, the cell amount with 1393B20 scaffolds was highest at day 1 of culture after which it decreased. However, with B12.5MgSr scaffolds, cell amount stayed constant.

1393B20 scaffolds showed higher cell density at the top and inside of the scaffolds, compared to B12.5MgSr scaffolds, at D1. At D3 and D7 the difference between the 2 glasses in this case is not significant as the cell density in 1393B20 scaffolds decreases, while the cell density increases for the B12.5MgSr BAG scaffolds. This decrease is most probably due to hADSCs reaching confluency at D1 which causes cells to be more sensitive to detachment.

These results indicate that, unlike the B12.5MgSr scaffolds, the 1393B20 scaffolds do not prevent cell viability and proliferation both at the top, inside and at the bottom of scaffolds. This is in accordance with results from live/dead assay.

#### Morphology

**Fig. 16:**
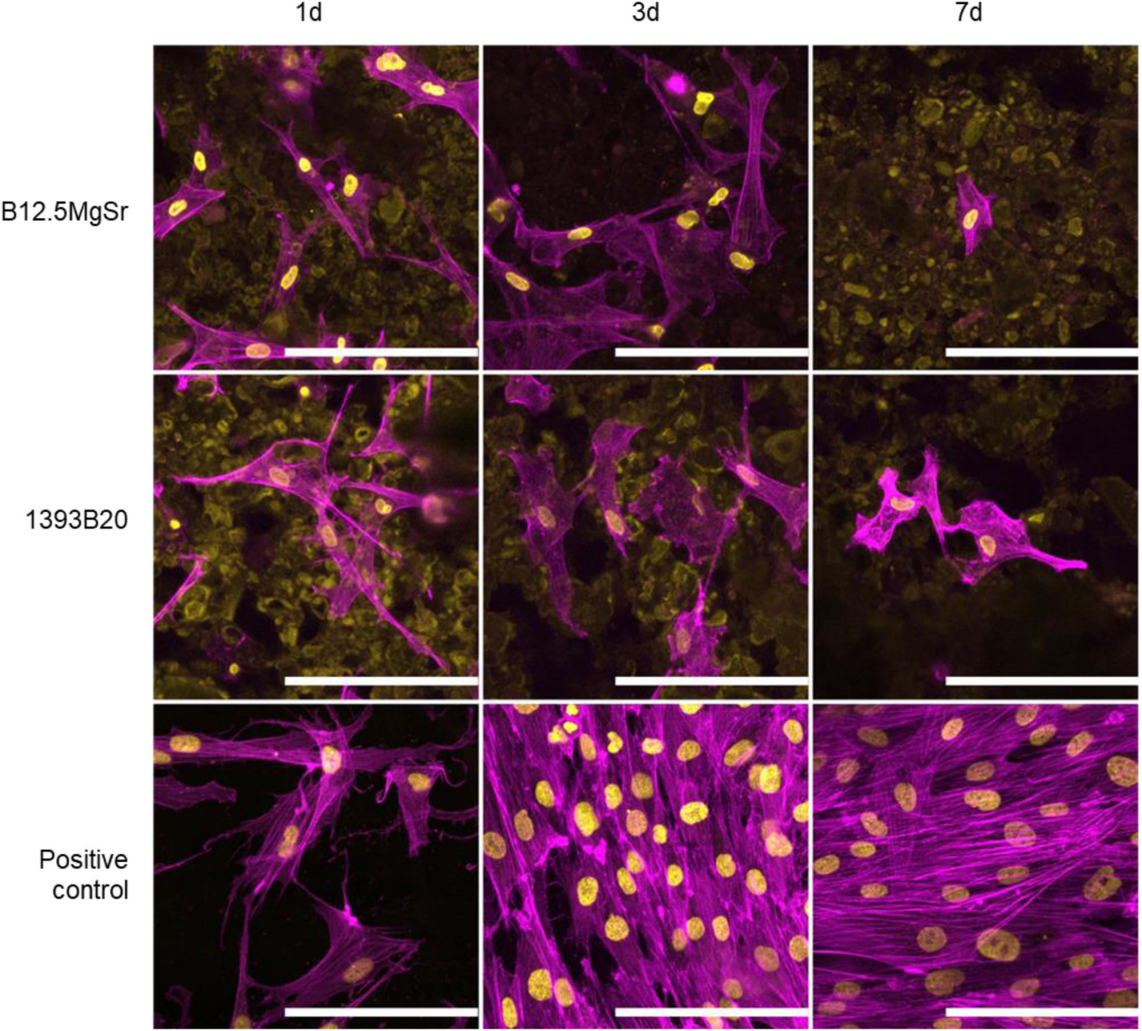
Morphology of hADSCs analysed by nuclei (DAPI, yellow) and cytoskeleton (phalloidin, magenta) cytochemical staining after 1, 3 and 7 days of culture in αMEM. Dim yellow staining is due to autofluorescence of the scaffold. Scale bar 100 µm

The morphology of hADSCs cultured on the top of the 1393B20 and B12.5MgSr BAG scaffolds was observed after 1, 3 and 7 days (Fig. 16).

The cells spread on both 1393B20 and B12.5MgSr scaffolds and their characteristic spindle-like morphology can be observed already after 1 day (Wu et al. 2016). There is no difference in the cytoskeleton of cells between 1393B20 and B12.5MgSr scaffolds. Cells spreading inside of the micropores on the surface of the materials can be observed due to autofluorescence of the scaffolds. These results further show that scaffolds do not present cytotoxic properties and support hADSCs attachment and spreading.

## Conclusions

Bioactive borosilicate 3D scaffolds, with and without dense top layer, were successfully prepared by robocasting. Scaffolds have large, interconnected pores (141–443 µm), with high overall porosity (38–46.5%) that meets the requirements for bone tissue engineering applications. The dense top was observed to introduce a gradient of porosity that mimics natural bone structure, limits soft tissue infiltration, and allow potential future membrane deposition.

Firstly, scaffolds were characterized by in vitro dissolution in TRIS solution (up to 10 weeks) and SBF (up to 2 weeks). 1393B20 scaffolds exhibited faster dissolution in TRIS but slower in SBF compared to B12.5MgSr scaffolds. The variation in the dissolution kinetics between TRIS and SBF is probably due to different rates of precipitation of HA in TRIS and SBF. Most importantly, the consumption of P^3-^ and Ca^2+^ ions during immersion in SBF suggests the formation of a HA-like layer, considered as the first sign of bioactivity. This was further confirmed by FTIR-ATR spectroscopy and SEM-EDX. Ultimately, there was no significant difference between scaffolds with and without dense top layer.

Next, the effect of preincubation time (48 h in TRIS + 24 h in αMEM) on burst ion release, which is known to be toxic to the cells, was investigated in the perspective of cell behavior studies. Preincubation was shown to significantly decrease burst release of ions.

Finally, the hADSCs behaviour in direct contact with the scaffolds was investigated, showing that the cells were able to proliferate and spread on the developed scaffolds while maintaining characteristic spindle morphology. 1393B20 allowed better cell viability compared to B12.5MgSr scaffolds. The B12.5MgSr decreased cell viability was assigned to the depletion of Ca and/or higher pH in cell culture medium compared to 1393B20. It is possible that toxicity of B12.5MgSr scaffolds could be decreased using dynamic cell culture, since agitation could also improve the transport of ions from inside to outside the scaffolds. This would lead to an averagely lower and more homogeneous ion concentration in the medium, which could be beneficial for cells.

In future, research will focus on 1393B20 scaffolds with gradient of porosity and its impact on hADSCs differentiation. To further improve the applicability of these constructs, combining them with persistent luminescence particles for enhanced bioimaging and in-vivo degradation tracking could be beneficial. Moreover, scaffolds performance in vivo should also be investigated.

## Supplementary information

Suplementary S1
