# Supplementary material for: Pore graded borosilicate bioactive glass scaffolds: in vitro dissolution and cytocompatibility
Source: J Mater Sci Mater Med. 2024 Mar 20;35(1):17. doi: 10.1007/s10856-024-06791-1 (PMC10954867; doi:10.1007/s10856-024-06791-1)
Supplement: Supplementary file 1 — Suplementary S1 [file 10856_2024_6791_MOESM1_ESM.docx]

Pore graded borosilicate bioactive glass scaffolds: in vitro dissolution and cytocompatibility.

Agata Szczodra^a^*, Amel Houaoui^a^, Turkka Salminen^b^, Markus Hannula^a^, Virginia Alessandra Gobbo^a^, Sonya Ghanavati^a^, Susanna Miettinen^a,c^, Jonathan Massera^a^

^a^Tampere University, Faculty of Medicine and Health Technology, Tampere, Finland

^b^Tampere University, Faculty of Engineering and Natural Sciences, Tampere, Finland

^c^ Research Services, Wellbeing Services County of Pirkanmaa, Tampere University Hospital, Tampere, Finland

* Corresponding author (Agata Szczodra, [agata.szczodra@tuni.fi](mailto:agata.szczodra@tuni.fi))


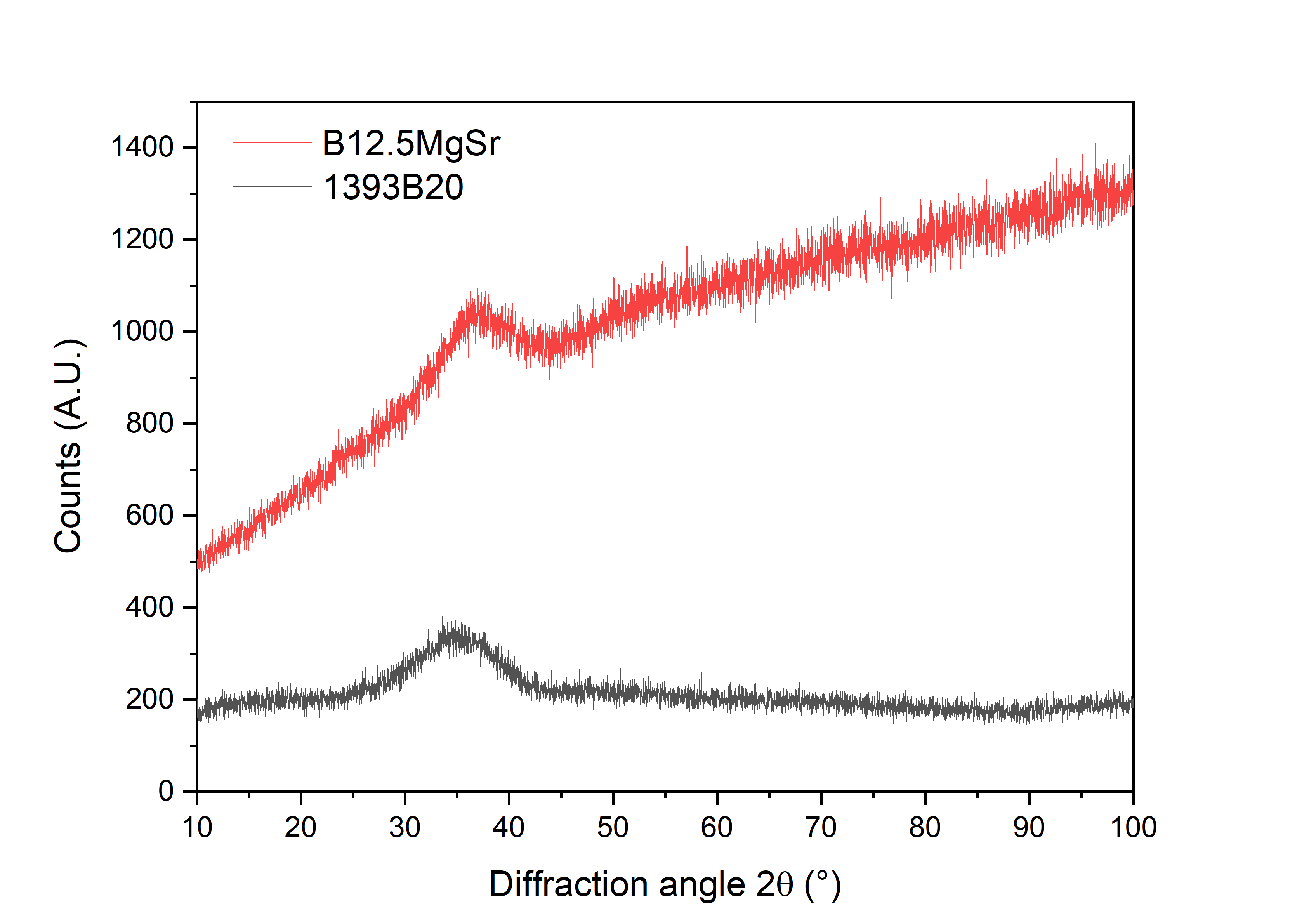
Crystallization is known to decrease or suppress bioactivity in bioactive glass scaffolds [1]. Thus, it is important to guarantee that the glass scaffolds remain amorphous after processing. Figure S1 presents the XRD pattern of the glass B12.5-Mg-Sr and 1393B20 post-sintering.

FIGURE S1: XRD pattern of B12.5-Mg-Sr and 1393B20 scaffolds post-sintering prepared via robocasting .

XRD spectra only exhibit broad hallow without visible diffraction peaks. These results confirm that post-sintering, no significant crystallization takes place and B12.5 and 1393B20 scaffolds stay amorphous.

1. Fabert M, Ojha N, Erasmus E, Hannula M, Hokka M, Hyttinen J, et al. Crystallization and sintering of borosilicate bioactive glasses for application in tissue engineering. J Mater Chem B. 2017;5:4514–25.
